# Leaf senescence: progression, regulation, and application

**DOI:** 10.1186/s43897-021-00006-9

**Published:** 2021-06-16

**Authors:** Yongfeng Guo, Guodong Ren, Kewei Zhang, Zhonghai Li, Ying Miao, Hongwei Guo

**Affiliations:** 1 Tobacco Research Institute, Chinese Academy of Agricultural Sciences, Qingdao, 266101 Shandong China; 2 Ministry of Education Key Laboratory for Biodiversity Science and Ecological Engineering, School of Life Sciences, Fudan University, Shanghai, 200438 China; 3 Institute of Plant Genetics and Developmental Biology, College of Chemistry and Life Sciences, Zhejiang Normal University, Jinhua, 321004 Zhejiang China; 4 Beijing Advanced Innovation Center for Tree Breeding by Molecular Design, Beijing Forestry University, Beijing, 100083 China; 5 Fujian Provincial Key Laboratory of Plant Functional Biology, Fujian Agriculture and Forestry University, Fuzhou, 350002 Fujian China; 6 Key Laboratory of Molecular Design for Plant Cell Factory of Guangdong Higher Education Institutes, Department of Biology, Southern University of Science and Technology (SUSTech), Shenzhen, 518055 Guangdong China

**Keywords:** Leaf senescence, Chlorophyll degradation, Phytohormones, Abiotic stress, Chromatin remodeling, Nutrient remobilization, Yield

## Abstract

Leaf senescence, the last stage of leaf development, is a type of postmitotic senescence and is characterized by the functional transition from nutrient assimilation to nutrient remobilization which is essential for plants’ fitness. The initiation and progression of leaf senescence are regulated by a variety of internal and external factors such as age, phytohormones, and environmental stresses. Significant breakthroughs in dissecting the molecular mechanisms underpinning leaf senescence have benefited from the identification of senescence-altered mutants through forward genetic screening and functional assessment of hundreds of *senescence-associated genes* (*SAGs*) *via* reverse genetic research in model plant *Arabidopsis thaliana* as well as in crop plants. Leaf senescence involves highly complex genetic programs that are tightly tuned by multiple layers of regulation, including chromatin and transcription regulation, post-transcriptional, translational and post-translational regulation. Due to the significant impact of leaf senescence on photosynthesis, nutrient remobilization, stress responses, and productivity, much effort has been made in devising strategies based on known senescence regulatory mechanisms to manipulate the initiation and progression of leaf senescence, aiming for higher yield, better quality, or improved horticultural performance in crop plants. This review aims to provide an overview of leaf senescence and discuss recent advances in multi-dimensional regulation of leaf senescence from genetic and molecular network perspectives. We also put forward the key issues that need to be addressed, including the nature of leaf age, functional stay-green trait, coordination between different regulatory pathways, source-sink relationship and nutrient remobilization, as well as translational researches on leaf senescence.

## Introduction

Senescence is the final stage of plant development and is characterized by a series of programmed disassembly and degenerative events (Guo and Gan 2005; Lim et al. 2007). In plants, there are two types of senescence: mitotic and post-mitotic senescence (Gan and Amasino 1997; Guo and Gan 2005). Mitotic senescence occurs in shoot apical meristem (SAM) containing multipotent stem cells, similar to replicative senescence in mammalian cell cultures and yeast (Gan and Amasino 1997; Guo and Gan 2005). In contrast, post-mitotic senescence occurs in organs such as leaves and flowers. Leaves are organs that characterize plants as autotrophic organisms and perhaps the primary source of food on earth which use light energy to fix carbon. As leaves age, chloroplast degeneration is initiated, paralleled by catabolism of macromolecules, including nucleic acids, proteins and lipids. The released nutrients are exported to other developing organs, such as new buds, young leaves, flowers or seeds, which leads to increased reproductive success (Lim et al. 2007). In perennial plants, such as deciduous trees, nutrients disassembled from senescent leaves are relocated to form bark storage proteins (BSP) in phloem tissues, stored over the winter, and then remobilized and reutilized for shoot or flower growth during the next growing season (Cooke and Weih 2005; Keskitalo et al. 2005). Therefore, the appropriate initiation and progression of leaf senescence are essential for plant fitness (Uauy et al. 2006). Efficient senescence is critical for maximizing viability in the next generation or season, while premature senescence induced by numerous environmental factors decreases the yield and fresh product quality of crop plants (Hortensteiner and Feller 2002). These insights suggest that leaf senescence evolves as a life history strategy and is of substantial biological significance. Understanding the regulatory mechanisms of leaf senescence will provide valuable clues and a theoretical basis for manipulation of this trait in agronomically important plants (Guo and Gan 2014).

Leaf senescence is not a passive but a highly coordinated process regulated by hundreds of *senescence-associated genes* (*SAG*s), whose transcripts increase as leaves age (Guo and Gan 2005; Lim et al. 2007). Many breakthroughs in dissecting the regulatory mechanisms underpinning leaf senescence have benefited from the identification and functional assessment of hundreds of SAGs and their corresponding mutants in *Arabidopsis thaliana*, tomato (*Solanum lycopersicon*), tobacco (*Nicotiana tabaccum*), rice (*Oryza sativa*) or wheat (*Triticum aestivum*) (Breeze et al. 2011; Woo et al. 2016; Li et al. 2018, 2020). Forward genetic studies by screening for mutants affected in senescence and reverse genetic analysis of SAGs provide insights into the molecular mechanisms of leaf senescence. Currently, 5853 SAGs and 617 mutants from 68 species have been identified and manually curated and extensively annotated, which facilitate the systematical and comparative investigation of leaf senescence (Li et al. 2020). It is now clear that leaf senescence is a highly complex genetic program strictly controlled by multiple layers of regulation, including transcriptional, post-transcriptional, translational and post-translational regulation (Woo et al. 2013, 2019). Moreover, the successful use of multi-omics methods has enabled the study of the complex process of leaf senescence, replacing the component-based static view with a network-based spatial-temporal understanding (Breeze et al. 2011; Guo 2013; Kim et al. 2018).

Leaf senescence is a genetically controlled developmental process (Gan and Amasino 1997; Lim et al. 2007; Kim et al. 2009). However, the initiation of leaf senescence is regulated by an array of external and internal signals that are integrated into the age information (Guo and Gan 2005; Lim et al. 2007). Plant hormones are major players influencing each stage of leaf senescence, including the initiation, progression and terminal phase of senescence. Ethylene, jasmonic acid (JA), salicylic acid (SA), abscisic acid (ABA), and strigolactones (SLs) promote leaf senescence, while cytokinins (CKs), gibberellic acid (GA), and auxin delay leaf senescence (Gan and Amasino 1995, 1997; Lim et al. 2007; Miao and Zentgraf 2007; Li et al. 2013; Zhang et al. 2013). Multiple environmental factors, including abiotic stresses such as drought, salt, DNA damage, high or low temperature, darkness and nutrient deficiency, and biotic stresses such as pathogen infection and phloem-feeding insects are also critical in regulating senescence (Lim et al. 2007; Guo and Gan 2012; Sade et al. 2018). Recent studies reveal that DNA damage, caused by endogenous insults or exogenous genotoxic stresses, might be one of the main determinants of leaf senescence (Li et al. 2020; Zhang et al. 2020c). Cellular calcium acts as a universal second messenger, which has allosteric effects on numerous enzymes and proteins in a variety of cellular responses. In plants, calcium signaling is evoked by endogenous and environmental factors. Ca^2+^ ions play an important role in plant senescence, and exogenous application of Ca^2+^ delays the senescence process of detached leaves (Poovaiah and Leopold 1973). Elevated CO_2_ usually leads to the accumulation of sugars and the decrease of nitrogen content in plant leaves, resulting in the imbalance of C/N ratio in mature leaves, which is also one of the main factors causing premature leaf senescence (Wingler et al. 2004; Agüera and De la Haba 2018). For plants, stress-induced premature senescence may not be a passive choice, but is an evolutionary fitness strategy, which speeds up the reproduction of the next generation under unfavorable living conditions (Sade et al. 2018). However, each factor does not work independently, but has mutual promotion or inhibition (Guo and Gan 2012). Environmental stress factors trigger the changes of endogenous hormones, and then affect leaf senescence through a complex regulatory network instead of a linear way.

Although continuously increasing efforts have been devoted to leaf senescence research, many issues remain to be addressed on this topic (Woo et al. 2013). When does leaf senescence start? What is the molecular nature of age? How are external signals integrated into the plant internal age information? Is the regulatory mechanism of leaf senescence conserved among different plant species? What is the general mechanism of leaf senescence? In this article, we review recent advances in understanding leaf senescence and longevity through molecular, genetic and network analyses. Although our review mainly focuses on *Arabidopsis* leaf senescence, we also discuss critical findings and translational research in agronomically important plants. Examples of genetic manipulation of leaf senescence for higher yield, better quality, or improved horticultural performance in crop plants are also discussed. Although the knowledge gained in the annual plant *Arabidopsis* may not be universally applicable to all plants, it will provide precious clues for dissecting regulatory mechanisms in other plants (Lim et al. 2007). It also provides the biological information required for developing high-quality and high-yield crops by finely tuning the senescence process (Gan and Amasino 1995; Rivero et al. 2007; Guo and Gan 2014).

## Chlorophyll degradation and chloroplast degeneration--hallmarks of leaf senescence

Leaf senescence is a degenerative process among which cellular organelles and biomolecules (including nucleic acids, proteins, and lipids) are broken down and the resultant catabolites are mobilized to sink tissues such as reproductive organs and new leaves. Chloroplasts possess approximately 70% of the total proteins in green leaves. As such, massive breakdown of chloroplasts during leaf senescence is pivotal for nitrogen and carbon remobilization (Kusaba et al. 2009). Moreover, coordinated degradation of chlorophyll (Chl) and its associated proteins during this process is crucial for detoxification, as free Chl and their catabolic intermediates upstream of primary fluorescent chlorophyll catabolites (pFCCs), are photosensitizers, which can cause reactive oxygen species (ROS) burst and subsequent cell damage and/or cell death (Mur et al. 2010; Hoertensteiner 2013). In this section, we summarize the biochemistry and regulation of Chl degradation. We also review our current understanding of chloroplast degeneration, with a focus on protein degradation and the cellular machinery involved.

### The biochemical pathway of chlorophyll degradation

**Fig. 1:**
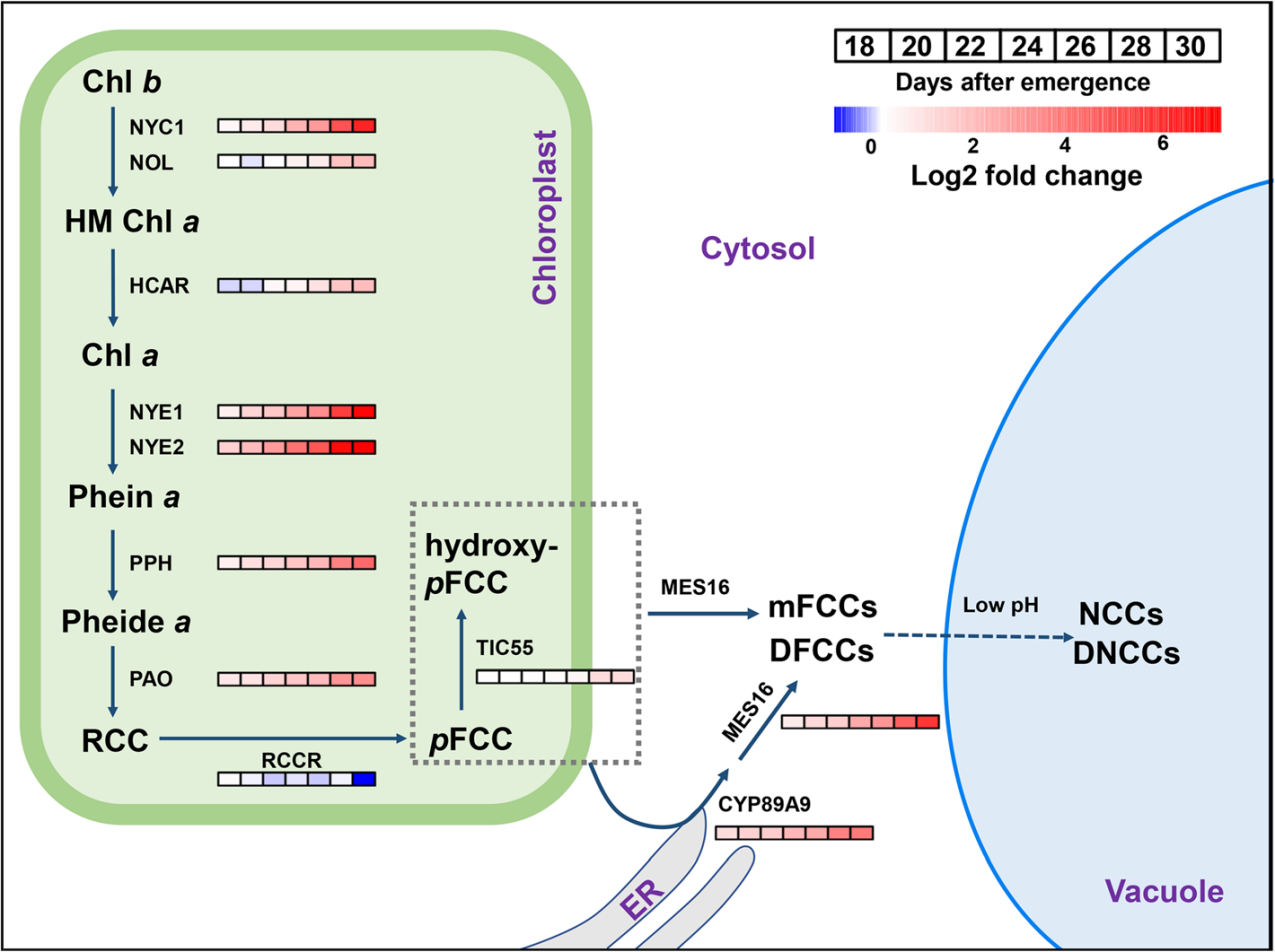
The biochemical pathway of Chl degradation. The initial steps of Chl catabolism occur in the Chloroplast. pFCC and hydroxy-pFCC are released to the cytosol by unknown mechanisms. Additional modifications are catalyzed by the ER-localized CYP89A9 and the cytosol MES16. mFCCs and DFCCs are imported into the vacuole and converted into NCCs and DNCCs under acidic conditions. Heatmaps show relative expression values of each gene at indicated days after emergence (DAE) versus that at 16 DAE. A published dataset (GSE43616) is used for analysis (Woo et al. 2016). HM Chl a, 7-hydroxymethyl Chl a; Phein a, pheophytin a; Pheide a, pheophorbide a; RCC, red Chl catabolite; pFCC, primary FCC; mFCC, modified FCC; DFCC, dioxobilin-type (type-II) FCC; NCC: nonfluorescent Chl catabolite; DNCC: dioxobilin-type (type-II) NCC.

Chl degradation brings the first visible sign of leaf senescence. Autumn leaf coloring of deciduous plants, mainly due to Chl breakdown, represents one of the most magnificent sceneries on earth. Chl is composed of a planar structure of a tetrapyrrole ring (porphyrin), which bears a magnesium atom in the center and a hydrophobic phytol tail on the side. Terrestrial plants utilize two types of Chl species (i.e., Chl a and Chl b) for photosynthesis. Chl b differs from Chl a by the presence of a formyl group at the C7 position instead of a methyl group (Chen 2014). By taking advantage of both forward and reverse genetic approaches, the stepwise enzymatic breakdown of Chl has been elucidated (Fig. 1). Enzymes and their corresponding genes responsible for Chl breakdown are generally termed Chl catabolic enzymes (CCEs) and Chl catabolic genes (CCGs), respectively.

Chl b has to be converted to Chl a before it can be channeled into the degradation pathway. Two sequential enzymatic reactions catalyze the conversion. NON-YELLOW COLORING 1 (NYC1) and NYC1-like (NOL), two Chl b reductases, catalyze the reduction of Chl b to 7-hydroxymethyl Chl a (Kusaba et al. 2007; Horie et al. 2009; Sato et al. 2009). 7-hydroxymethyl Chl a is further reduced by the 7-hydroxymethyl Chl a reductase HCAR to produce Chl a (Meguro et al. 2011). In *Arabidopsis*, only *NYC1* is induced during leaf senescence, and its dysfunction results in Chl b over-accumulation (Horie et al. 2009; Sakuraba et al. 2012). However, NYC1 and NOL are both required for Chl b degradation in rice (Sato et al. 2009). 7-hydroxymethyl Chl a is barely detectable due to high expression of *HCAR* (Meguro et al. 2011). Chl a degradation is initiated *via* removing the central magnesium by the Mg-dechelatase NON-YELLOWINGs/ STAY-GREENs (NYEs/SGRs), which produces pheophytin a (Shimoda et al. 2016). NYE1 and NYE2 predominantly extract Mg from Chl a, whereas SGR-LIKE (SGRL), a distant paralogous protein of NYE1/2, is mainly expressed in non-senescent leaves and prefers chlorophyllide a as its substrate (Shimoda et al. 2016). Notably, malfunctions in NYEs and their orthologous genes results in stay-green phenotypes in diverse plant species, including Mendel’s green-cotyledon pea (Armstead et al. 2007; Jiang et al. 2007; Park et al. 2007; Ren et al. 2007; Barry et al. 2008; Hoertensteiner 2009; Zhou et al. 2011a; Christ and Hortensteiner 2014). Pheophytin a is hydrolyzed by the pheophytinase PPH to generate pheophorbide a and phytol (Schelbert et al. 2009). The porphyrin ring of pheophorbide a is then opened by the pheophorbide an oxygenase PAO to produce a red chlorophyll catabolite (RCC) (Pruzinska et al. 2003), while phytol has been implicated as a source for vitamin E biosynthesis (Zhang et al. 2014). RCC is further reduced into a primary fluorescent chlorophyll catabolite (pFCC) by the RCC reductase RCCR (Pruzinska et al. 2007). Additional modifications such as C32 hydroxylation, C1 deformylation and O84 demethylation, as catalyzed by TIC55, CYP89A9 and MES16, respectively, can occur on pFCC to produce diverse types of modified pFCCs and/or FCCs (Christ et al. 2012, 2013; Hauenstein et al. 2016). FCCs are further isomerized to non-fluorescent chlorophyll catabolites (NCCs) under low pH condition, which likely takes place in the vacuole (Oberhuber et al. 2008). NCCs have antioxidant properties that may play a role in maintaining the integrety of senescing cells (Mueller et al. 2007). Since the cleavage of the porphyrin ring is believed to be a landmark event during Chl degradation and the resultant linear catabolic intermediates are called phyllobilins, this Chl degradation pathway is referred to as the “PAO/phyllobilin” pathway or simply as the PAO pathway (Kuai et al. 2018).

### Regulation of Chl degradation

Chl degradation occurs not only during specific developmental windows such as leaf senescence, fruit ripening, and seed maturation, but also under unfavorable environmental conditions including various biotic and abiotic stresses (Kuai et al. 2018). Therefore, *CCGs* should be able to be induced by diverse signals. On the other hand, to avoid phototoxicity, CCEs must act cooperatively to degrade Chl timely and efficiently. To achieve this, *CCGs*, including *NYE1*, *PPH* and *PAO*, are strongly co-expressed. In fact, searching for genes co-regulated with *NYE1* has led to the identification of CRN1 (Co-regulated with NYE1)/PPH (Ren et al. 2010). Recent advances in the transcriptional regulation of *CCGs* highlight the importance of key transcription factors (TFs) downstream of ethylene (Qiu et al. 2015), ABA (Sakuraba et al. 2014; Gao et al. 2016), JA (Zhu et al. 2015), and light (Song et al. 2014; Zhang et al. 2015; Chen et al. 2017a) signaling pathways in direct induction of *CCGs* expression.

During ethylene-mediated leaf senescence, ETHYLENE-INSENSITIVE3 (EIN3), its target TF ORESARA 1 (ORE1)/ANAC092, and the ethylene biosynthesis gene *ACS2* form a positive feedback loop to amplify upstream signals. On the other hand, EIN3, ORE1, and *CCGs* (including *NYE1*, *NYC1*, and *PAO*) form a coherent feedforward loop to stimulate *CCGs* expression (Qiu et al. 2015). Similarly, MYC2/3/4, ANAC019/055/072, and several *CCGs* also forms a coherent feedforward loop during JA-mediated leaf senescence (Zhu et al. 2015). Moreover, ABA INSENSITIVE 5 (ABI5), ENHANCED EM LEVEL (EEL), and ABA RESPONSIVE ELEMENTS-BINDING FACTOR 2/3/4 directly activate multiple *SAGs* expression (including *CCGs*) downstream of the ABA signaling pathway (Sakuraba et al. 2014; Gao et al. 2016). Intriguingly, Chl degradation during seed maturation largely depends on another ABA-pathway TF ABI3. *ABI3* is highly expressed in seeds and directly activates *NYE1* and *NYE2* expression during seed maturation. Like the *nye1 nye2* double mutant, the *abi3* mutant produces mature green seeds (Delmas et al. 2013). Prolonged darkness treatment has been widely used as a tool for studying leaf senescence. PHYTOCHROME-INTERACTING FACTOR (PIF) family proteins are key TFs in the light signaling pathway, which are stabilized in the nucleus under dark conditions (Sanchez et al. 2020). PIF4/5 can directly or indirectly (through ABI5/EEL, EIN3, or ORE1) activates *CCGs* expression during dark-induced leaf senescence. These TFs may also act through forming multiple coherent feedforward loops (Sakuraba et al. 2014; Song et al. 2014). Moreover, a MADS-box TF SOC1 negatively regulates *NYE1* and *PPH* expression (Chen et al. 2017a). In rice, OsNAC2 and OsNAP (NAC-LIKE, ACTIVATED BY AP3/PI) directly target *CCGs* and several *SAGs* during ABA- and age-dependent leaf senescence (Liang et al. 2014; Mao et al. 2017). In *Citrus sinensis*, the ethylene-responsive factor CitERF13 promotes Chl degradation likely through inducing the expression of *PPH* (Yin et al. 2016). Recently, the maize TF ZmNAC126 is reported to function downstream of the ethylene signaling pathway and activate the expression of *CCGs* *via* directly binding to their promoters to accelerate leaf senescence (Yang et al. 2020).

Transcription of *CCGs* is also subject to epigenetic regulation. He et al. (2018) identified a natural DNA methylation variation locus NMR19 (naturally occurring DNA methylation variation region 19–4, caused by insertion of LINE1 retrotransposon) at the *Arabidopsis PPH* promoter. DNA methylation levels of NMR19–4 correlates with suppressed *PPH* expression and retarded Chl degradation. More intriguingly, association analysis of 137 *Arabidopsis* accessions revealed that NMR19–4 may be involved in local climate adaptation (He et al. 2018). Recently, it was found that the H3K27me3 (trimethylation of lysine 27 on histone H3 protein) demethylase REF6 positively regulates the de-repression of *CCGs* and other *SAGs* through removing the silent chromatin marker H3K27me3 on respective genes (Wang et al. 2019). Altogether, these findings suggest that *CCGs* are critical convergent nodes for incoming signals during leaf senescence. The molecular relationship and crosstalk among TFs and their interactions with epigenetic factors on *CCGs* expression remain poorly understood.

CCEs are also subject to post-translational regulation. In *Brassica napus*, de-phosphorylation of PAO contributes to its increased activity during seed maturation (Chung et al. 2006). Recently, Xie et al. (2019) identified a conserved C-terminal cysteine-rich motif of NYE1 that is critical for its oligomerization through both inter- and intra-molecular disulfide bonds. In another study, BALANCE of CHLOROPHYLL METABOLISM 1 (BCM1), a CAAX-type endopeptidase, is highly abundant in non-senescent leaves and degrades NYE1 through physical interaction, providing a mechanism for repressing leaky expression of *NYE1*. During leaf senescence, *BCM1* is down-regulated and releases its inhibition on NYE1 accumulation (Shin et al. 2020).

Analyses of *CCG* mutants also point to a feedback mechanism, which possibly involves chloroplast-to-nucleus retrograde signaling. In the *nye1* and *nye1 nye2* mutants, not only Chl a but also Chl b is over-accumulated (Ren et al. 2007; Wu et al. 2016a). In accordance with this, overexpression of *NYE1* accelerates Chl b degradation through induction of *NYC1* expression (Sato et al. 2018). The *pph* and *pao* mutants only accumulate a tiny amount of their respective substrates but heavily retain Chl a and Chl b (Pruzinska et al. 2005; Schelbert et al. 2009). Transcriptomic analyses on three *CCG* mutants (i.e., *nye1, pph*, and *pao*) revealed that JA biosynthesis and signaling genes are preferentially induced in the *pao* mutant 2 days after dark treatment. Accordingly, JA production is enhanced in the *pao* mutant. The results suggest that pheophorbide a, the substrate of PAO, may serve as a feedback signal for JA production (Aubry et al. 2020). Not come singly but in pairs, DEX (dexamethasone)-induced overexpression of *NYE1* also stimulates JA production, which likely contributes to *SAGs* (including other *CCGs*) induction and consequently leaf senescence (Sato et al. 2018; Ono et al. 2019). Since the DEX induction system can induce ultrahigh expression levels of target genes in a very short time, it is tempting to speculate that a burst of NYE1 may transiently over-produce Chl catabolic intermediates including pheophorbide a. It is unknown whether pheophorbide a can act as a retrograde signal as several Chl biosynthetic intermediates do (Chi et al. 2013).

Plants have evolved a tightly coupled degradation mechanism of Chl and its binding proteins. In senescing leaves of *nye1*, *nye1 nye2* and *pph* mutants, not only Chls, but also their binding proteins (e.g., LHCA and LHCB proteins), are retained. Strikingly, such coupling mechanism appears to not exist during seed maturation (Li et al. 2017). The underlying mechanisms for coupling and uncoupling await investigation. Moreover, timely removal of high toxic catabolites is crucial. In *pao* and *rccr* mutants, over-accumulation of respective substrate catabolites (i.e., pheophorbide a and RCC) causes accelerated cell death phenotypes (Pruzinska et al. 2003, 2007).

### Chloroplast degradation

Chloroplast degeneration and degradation involve both plastidic and extraplastidic pathways (Otegui 2018; Buet et al. 2019; Zhuang and Jiang 2019). Although a number of chloroplast proteases are up-regulated during leaf senescence, only a few of them have been functionally characterized in senescence-associated protein degradation (Roberts et al. 2012). In tobacco, the chloroplast aspartic protease CND41 degrades Rubisco in vitro. The CND41 activity is enhanced in senescent leaves. Transgenic analyses suggest that CND41 positively regulates leaf senescence and Rubisco degradation (Kato et al. 2004, 2005). Similar conclusions are obtained in *Arabidopsis* under low N-induced leaf senescence (Diaz et al. 2008). A recent study showed that the barley cysteine protease HvPAP14 accumulates in senescent leaves and degrades multiple chloroplast proteins, including LHCB1/5, PSBO, and RbcL (Frank et al. 2019). Zelisko et al. (2005) reported that the metalloprotease FtsH6 is up-regulated during leaf senescence and is responsible for Lhcb3 degradation in *Arabidopsis*. However, no significant differences between *ftsh6* mutants and wild-type plants are reported in another study (Wagner et al. 2011). While it is generally accepted that plastidic proteases participate in early stages of chloroplast protein degradation, they mainly serve as quality control machinery in protein homeostasis (Van Wijk 2015). On the other hand, growing bodies of evidence suggest the existence of multiple extraplastidic trafficking vesicles for bulk degradation of chloroplast components, which are either autophagy-related proteins (ATG)-dependent or independent. The ATG-dependent vesicles include Rubisco-containing bodies (RCB, around 1 μm in diameter, sic passim) (Ishida et al. 2008), ATI1-GFP labels plastid-associated bodies (ATI-PS body, around 1 μm) (Michaeli et al. 2014), small starch granule-like structures (SSTG, < 0.5 μm) (Wang et al. 2013), and chlorophagy bodies (i.e. autophagic bodies containing an entire damaged chloroplast) (Izumi et al. 2017; Nakamura et al. 2018). The ATG-independent vesicles include senescence-associated vacuoles (SAVs, 0.8–1 μm) (Otegui et al. 2005; Martinez et al. 2008; Carrion et al. 2013) and CHLOROPLAST VESICULATION (CV)-containing vesicles (CCVs) (Wang and Blumwald 2014). These trafficking vesicles are eventually internalized into central vacuoles for degradation. The ubiquitin-26S proteasome system (UPS) is the major pathway for nuclear and cytosolic protein degradation in eukaryotes, but is absent inside of chloroplasts. However, cumulative evidence suggests the involvement of the UPS in chloroplast protein degradation. PUB4, a cytoplasmic-localized U-box E3 ubiquitin ligase, participates in the clearance of damaged chloroplasts. Loss of function in PUB4 results in precocious leaf senescence (Woodson et al. 2015). Moreover, the chloroplast-associated protein degradation (CHLORAD) system, which includes SP1 [a chloroplast outer-envelope-membrane (OEM) targeted RING-type ubiquitin E3 ligase], SP2 (another OEM-targeted Omp85-type protein) and CDC48 (a cytosol AAA-type ATPase), promotes leaf senescence likely by altering the chloroplast proteome through selective breakdown of translocon outer membrane complex (TOC) proteins (Ling et al. 2012, 2019). While neither PUB4 nor SP1 is involved in autophagic chloroplast degradation, combined mutations of PUB4 and AUTOPHAGY5 (ATG5) or ATG7 significantly accelerates leaf senescence under both natural and dark-induced conditions, suggesting synergistic interactions between the PUB4-associated UPS pathway and the autophagy pathway in protein degradation during leaf senescence (Kikuchi et al. 2020).

## Genetic control of leaf senescence

**Fig. 2:**
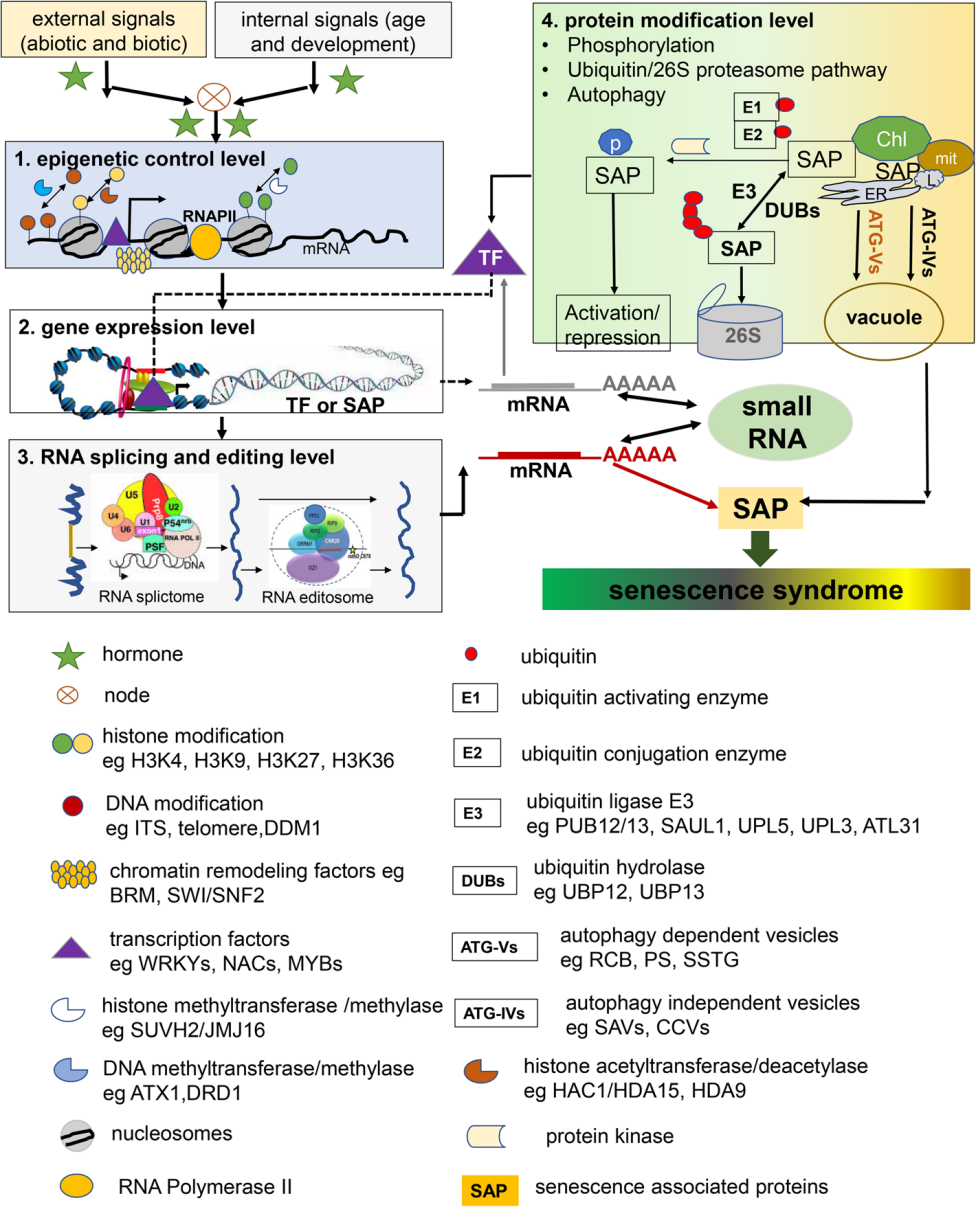
Multilayer-control of plant aging and senescence. Leaf senescence involves highly complex genetic programs that are tightly tuned by multiple layers of regulation, including DNA methylation, histone methylation/acetylation and chromatin remodeling, transcription regulation by WRKYs, NACs and MYBs TFs, as well as post-transcriptional regulation including miRNAs, RNA splicing and RNA editing, translational, and post-translational regulation including protein phosphorylation, ubiquitination and autophagy

The leaf senescence syndrome, including chloroplast/Chl degradation and other degenerative processes, involves highly complex genetic programs that are tightly tuned by multiple layers of regulation, including chromatin remodeling and transcription regulation, as well as post-transcriptional, translational, and post-translational regulation (Fig. 2).

### Epigenetic regulation

The structural dynamics of chromatin through histone modification and chromatin-remodeling enzymes is a key mechanism of leaf senescence regulation. After Humbeck’s group found that histone modification is altered during plant senescence (Ay et al. 2009), the genome-wide landscape of histone modification during developmental leaf senescence in *Arabidopsis* has been explored by combining chromatin immunoprecipitation-sequencing (ChIP-seq) and RNA-seq approaches. Two active marks, trimethylation of lysine 4 on histone H3 protein (H3K4me3) and acetylation of lysine 9 on histone H3 protein (H3K9ac), were identified to be associated with leaf senescence. H3K4me3 is relatively dominant compared with H3K9ac, and a subset of genes differentially expressed during leaf senescence is significantly correlated with the level of H3K4me3 (Brusslan et al. 2015). The first piece of evidence for a direct link between histone modification and control of leaf senescence comes from a study of upstream transcriptional regulation of several regulators of leaf senescence during plant aging. The suppressor of variegation 3–9-homologous2 (SUVH2) histone methyltransferase is involved in H3 lysine methylation and functions to delay leaf senescence (Ay et al. 2009). The JmjC-domain containing protein 16 (JMJ16) H3K4 demethylase on the other hand, functions as a senescence-promoting factor whose loss-of-function mutants show delayed senescence phenotypes and less enriched H3K4me3 in the promoter of the positive senescence regulator *WRKY53* (Liu et al. 2019). Both H3K27me3 demethylase REF6 (Wang et al. 2019) and HISTONE DEACETYLASE9 (HDA9) (Chen et al. 2016) promote leaf senescence: REF6 functions through directly activating positive senescence regulators such as ETHYLENE INSENSITIVE 2 (EIN2), ORE1, and NAP, while HDA9 forms a complex with POWERDRESS and WRKY53, and WRKY53 directs POWERDRESS and HDA9 to W-box containing promoters of negative senescence regulators, including AUTOPHAGY 9 (ATG9), NUCLEAR PROTEIN X 1 (NPX1), and WRKY57. HDA15 interacts with the single-stranded DNA-binding protein WHIRLY1 and affects H3K9ac enrichment in the promoter of *WRKY53* to suppress its transcription, thus delaying leaf senescence (Huang et al. 2018). Moreover, histone acetyltransferase HAC1 has been shown to promote leaf senescence (Hinckley et al. 2019), while several HDACs, including HDA9, HDA15, HD2C, and AtSRT1, negatively regulate stress-induced senescence in *Arabidopsis* (Hu et al. 2019b; Ueda and Seki 2020).

The importance of chromatin-mediated regulation in leaf senescence has also been inferred from mutation analyses of several chromatin-remodeling factors, including DEFECTIVE IN RNA-DIRECTED DNA METHYLATION 1 (DRD1), and DECREASED DNA METHYLATION 1 (DDM1) (Cho et al. 2016). Loss-of-function mutants of DRD1 or DDM1 exhibit delayed leaf senescence while dysfunction mutation of BRM accelerates leaf senescence (Efroni et al. 2013; Li et al. 2016; Xu et al. 2016a; Archacki et al. 2017). DEMETER-like DNA demethylase gene *DML3* is specifically expressed during leaf senescence. Knockout of DML3 results in DNA hypermethylation in the promoters of many *SAG*s whose expression is consequently suppressed, leading to a significant delay in leaf senescence. This suggests that DML3-mediated DNA demethylation regulates leaf senescence by controlling the expression of a subset of *SAG*s (Yuan et al. 2020). However, the detailed mechanisms of how histone modification and chromatin-remodeling enzymes directly regulate *SAG*s expression remain exclusive.

### Transcriptional regulation

Time-course gene-expression profiling of *Arabidopsis* leaves during aging indicated that 10–16% of genes show expression changes during leaf senescence (Breeze et al. 2011; Woo et al. 2016). The importance of TF-mediated transcriptional regulation has emerged from the identification of master TFs that play critical roles in the leaf senescence program.

Master TFs are crucial for regulating the temporal expression of *SAGs* during leaf aging (Penfold and Buchanan-Wollaston 2014; Huang et al. 2015; Kim et al. 2016b, 2018). Among the TF families, the WRKY family members involved in leaf senescence include WRKY6, WRKY53, WRKY54, WRKY22, WRKY70, and WRKY75 (Robatzek and Somssich 2001; Miao et al. 2004; Zentgraf et al. 2010; Zhou et al. 2011b; Besseau et al. 2012; Guo et al. 2017). The NAC family is one of the largest gene families in plants and plays a central role in regulating leaf senescence. More than 50% of the NAC family genes show expression changes during leaf aging in *Arabidopsis*. Genetic studies identified a number of NACs as positive (ANAC016, AtNAP, ORS1, and ORE1) or negative (JUB1 and AVNI2) regulators of leaf senescence (Wu et al. 2012a; Hickman et al. 2013; Kim et al. 2016b, 2018). Members in the NAC family and the WRKY family may interact with each other by activating or repressing transcription or by forming protein complexes in regulating the expression of downstream genes (Zentgraf et al. 2010; Besseau et al. 2012; Kim et al. 2016b, 2018). The basic helix-loop-helix (bHLH) TFs also coordinate in regulating leaf senescence. The IIIe subgroup bHLH TFs MYC2, MYC3, and MYC4 function redundantly to activate JA-induced leaf senescence in which MYC2 binds to and activates the promoter of target gene *SAG29*. The IIId subgroup bHLH factors bHLH03, bHLH13, bHLH14, and bHLH17, on the other hand, bind to the promoter of *SAG29* and repress the MYC2-activated expression of *SAG29* to attenuate JA-induced leaf senescence (Qi et al. 2015). Furthermore, evidence of MYBs’ involvement in leaf senescence is increasing (Jaradat et al. 2013; Huang et al. 2015; Qi et al. 2015; Goossens et al. 2017).

### Post-transcriptional regulation

Key regulators of leaf senescence are also subject to multiple post-transcriptional level modulations, including RNA editing, splicing, transport, and degradation by miRNAs and non-coding RNAs binding to cis-elements in mRNA.

Weigel’s group firstly reported that *miR319* regulates leaf senescence *via* repressing the expression of several members of the TEOSINTE BRANCHED/CYCLOIDEA/PCF (TCP) family TFs, which function in promoting leaf senescence in *Arabidopsis* (Schommer et al. 2008). Then Nam’s group reported that *miR164* controls *ORE1* transcription and leaf aging (Schommer et al. 2008; Kim et al. 2009; Li et al. 2013). In genome-wide studies of miRNA abundances during leaf development, more than 50 miRNAs show differential expression during senescence. Predicted target genes of the senescence-regulated miRNAs are involved in stress response, plant hormones, nutrient mobilization and cell structural integrity (Thatcher et al. 2015; Woo et al. 2016). When miRNA-dependent gene regulatory networks were investigated in maize and rice (Xu et al. 2014; Wu et al. 2016b), common miRNA species, including *miR159*, *miR160*, *miR167* and *miR172*, were identified to be senescence-regulated, suggesting conserved functionality of these miRNAs in senescence regulation (Yolcu et al. 2017).

In addition to miRNA, a few other types of small RNAs (smRNAs) have been identified to be regulated by leaf senescence. Among these, 200 AGO1-enriched smRNA–target *SAG* pairs (Qin et al. 2015), 117 trans-acting small interference RNAs (tasiRNAs)–target *SAG* pairs, and 235 21-nt smRNA–target *SAG* pairs (Woo et al. 2016) have been identified to be potentially involved in leaf senescence. However, only a small number of miRNAs, including *miR840*, *miR585*, and *miR172*, have been characterized to regulate leaf senescence *via* direct cleavage and/or translational repression of target *SAG* genes (Ren et al. 2020; Wu et al. 2020).

Other than smRNAs components of spliceosome and editosome are also part of the post-transcriptional regulatory mechanisms underlying leaf senescence. A minor spliceosome component U11-48 K, which is required for correct splicing of U12 introns, is required for normal plant development, including leaf senescence and cell fate (Xu et al. 2016a; Gault et al. 2017; Bai et al. 2019). Alternative splicing (AS) is an important factor in gene regulation and gene splicing. It is involved in a variety of plant growth and developmental processes, such as induction of flowering (Slotte et al. 2009), plant responses to changing environmental conditions, pathogen attacks (Barbazuk et al. 2008), as well as leaf senescence (Riester et al. 2019). ETHYLENE RESPONSE FACTOR4 (ERF4), a positive regulators of leaf senescence, functions together with ERF8 in suppressing the expression of its direct target gene *EPITHIOSPECIFIER PROTEIN/ EPITHIOSPECIFYING SENESCENCE REGULATOR* (*ESP/ESR*), negatively regulating the transcription factor WKRY53 and delayed leaf senescence (Koyama et al. 2013; Miao and Zentgraf 2007). Alternative splicing and polyadenylation of ERF4 result in two ERF4 isoforms: one containing the EAR-motif (ERF4-R), one lacking it (ERF4-A) (Lyons et al. 2013). ERF4-A acts as a transcriptional activator and ERF4-R as a repressor of their direct target gene *CATALASE3* (*CAT3*), controlling the concentrations of ROS in cells and regulating leaf senescence (Riester et al. 2019). The editosome core component MORF9, which is required for correct editing of *RPS14–80* and *RPS14–149*, also affects leaf senescence (Woo et al. 2006; Hackett et al. 2017; Sun et al. 2018; Tian et al. 2019). Further studies are needed to uncover the potential roles of other types of post-transcriptional mechanisms involving mRNA processing (5′-capping, splicing, and 3′-end processing), mRNA modification, and mRNA export machinery.

### Post-translational regulation

Post-translational regulation is crucial in cellular signaling. During leaf senescence, although phosphorylation, glycosylation and other protein modifications might be as important (Ahmad and Guo 2019), the predominant form of post-translational regulation that has been well-characterized is protein degradation, which is critical not only in signaling transduction, but also for the execution of the senescence syndrome. The main protein degradation routes during plant senescence are proteasome and autophagy, which, together, enable turnover of organelles and aberrant or aggregated proteins, proper nutrient recycling, and precise control of the homeostasis of senescence regulators. The UPS involves the covalent attachment of multiple ubiquitins to selected target proteins, triggering their recognition and degradation by the 26S proteasome (Vierstra 2009). Whereas the UPS typically removes single proteins, autophagy removes protein complexes, protein aggregates, part of or even whole organelles (Schreiber and Peter 2014). Autophagy appears to prevent senescence, while the proteasome functions as a positive regulator of senescence (Wang and Schippers 2019). However, interestingly, current evidence described above (2.3) suggests synergistic interactions between the 26S ubiquitin-proteasome and the autophagy pathway in chloroplast protein degradation during leaf senescence (Kikuchi et al. 2020).

Initiation of leaf senescence leads to upregulation of ATG genes (Masclaux-Daubresse et al. 2014). Disruption of different ATGs, including ATG2, ATG4a/4b, ATG5, ATG7, ATG8, ATG9, ATG10, and ATG18a, causes defective autophagy and precocious leaf senescence in *Arabidopsis*, especially under nutrient-limited conditions (i.e., low nitrate) (Minina et al. 2018; Wang and Schippers 2019). Although autophagy has long been thought to be a synonym for nonselective bulk protein degradation, an increasing number of studies have demonstrated selective autophagic-dependent degradation of cell components, including mitochondria (Li et al. 2014), peroxisomes (Shibata et al. 2013), chloroplasts (Izumi et al. 2017), ribosomes (Hillwig et al. 2011), the proteasome (Marshall et al. 2015), and the endoplasmic reticulum (Yang et al. 2016). For instance, ATG8-INTERACTING PROTEIN1 (ATI1) is involved in autophagy-dependent vesicular trafficking of chloroplast proteins to the vacuole, and selective breakdown of mitochondria-resident proteins and mitochondrial vesicles during leaf senescence (Li et al. 2014; Michaeli et al. 2014). While most studies support the role of autophagy in preventing senescence, ATG8, through its interaction with the multidrug and toxic compound extrusion (MATE) transporter ABNORMAL SHOOT3 (ABS3), promotes senescence and protein degradation at late endosome (Jia et al. 2019). These data suggest that the components of autophagy may have dual roles (executioner and procrastinator) in the onset and progression of senescence to maximize the salvaging of remaining nutrients.

In contrast with the global upregulation of *ATG* genes, only a fraction of the proteasome subunit genes increases their expression during senescence (Guo and Gan 2012). In senescing leaves of oilseed rape, barley, and *Arabidopsis*, the proteasome is highly active (Poret et al. 2016; Velasco-Arroyo et al. 2016). Knocking down/out subunit genes of the proteasome in *Arabidopsis* often causes a delay in the onset of senescence (Lin et al. 2011). Loss of the regulatory particle subunit RPN10 significantly delays senescence, while the overexpression of *RPN5a* causes premature senescence (Book et al. 2009). In addition, application of proteasome inhibitors is capable of delaying the onset of senescence (Pak and Van Doorn 2004). Ubiquitination/ deubiquitination controlling ubiquitin dynamics in cells is one of the most common post-translational modifications (PTMs) and is involved in a wide repertoire of biological processes in plants. A set of enzymes and their targets in the ubiquitination cascade function as regulators of leaf senescence (Shu and Yang 2017). ORE9, an F-box protein, functions as a positive regulator of leaf senescence (Woo et al. 2001). The U-box (PUB) E3 ubiquitin ligases PUB12 and PUB13 negatively regulate stress-induced leaf senescence (Zhou et al. 2015). PUB44, also known as SENESCENCE-ASSOCIATED E3 UBIQUITIN LIGASE 1 (SAUL1) and NOT ORESARA 1 (NORE1), integrally mediates signals from temperature- and humidity-dependent defense programs and leaf senescence (Vogelmann et al. 2012). A RING-type ubiquitin ligase, ARABIDOPSIS TOXICOS EN LEVADURA 31 (ATL31), plays an important role in leaf senescence under high-CO2/ low-N conditions (Aoyama et al. 2014). WRKY53 directly activates ATL31 in response to the cellular C/N status of the plant, integrating the control of primary metabolism into leaf senescence (Aoyama et al. 2014).

Members of another HECT-type ubiquitin ligase E3 family (UPL1-UPL7) are also involved in leaf senescence (Lan and Miao 2019). Knocking-out UPL5 causes WRKY53 accumulation and early leaf senescence (Miao and Zentgraf 2010).

In contrary to the more intensively studied function of E3 ligases, insights into the specific roles of deubiquitination enzymes (DUBs) in leaf senescence are only recently emerging. Ubiquitin-specific proteases (UBPs) are the largest subfamily of DUBs with diverse functions in plants (Zhou et al. 2017). UBP12 and UBP13 have deubiquitinating activities and the *ubp12*-mild or the *ubp12ubp13* double mutants display altered flowering time, changes in the circadian rhythms and leaf senescence phenotype (Cui et al. 2013). UBP12 and UBP13 also accelerate leaf senescence by deubiquitinating and consequently stabilizing ORE1 (Park et al. 2019). Therefore, the ubiquitin dynamics in the cells is important for maintain protein functions during plant development.

Taken together, it seems that autophagy and the proteasome may differentially influence aging and the onset of senescence. Interactions between these two pathways in regulating senescence also exist. For example, as the proteasome can be removed by autophagy (Havé et al. 2018), a potential accumulation of proteasomes might be responsible for the early senescence phenotype of the autophagy mutants. It might also represent a type of compensation for the decreased proteolytic activities that occur.

In addition to protein degradation, senescence-regulating proteins are subject to a variety of PTMs. A total of 207 PTMs have been detected on SAG proteins in *Arabidopsis* from the Plant PTM Viewer database (Willems et al. 2019), including lysine acetylation, lysine methylation, lysine SUMOylation, lysine ubiquitination, O-GlcNAcylation, phosphorylation, reversible cysteine oxidation, and so on (Wang and Schippers 2019). Among them, phosphorylation might be highly relevant during the onset of senescence, as mitogen-activated kinase cascades have been shown to affect the timing of the senescence process in *Arabidopsis* (Zhou et al. 2009; Zentgraf et al. 2010; Xiao et al. 2015; Ren et al. 2017). In all cases, a better understanding of the role of PTMs in the senescence-associated proteins during plant senescence is needed.

## Hormonal regulation of leaf senescence

**Fig. 3:**
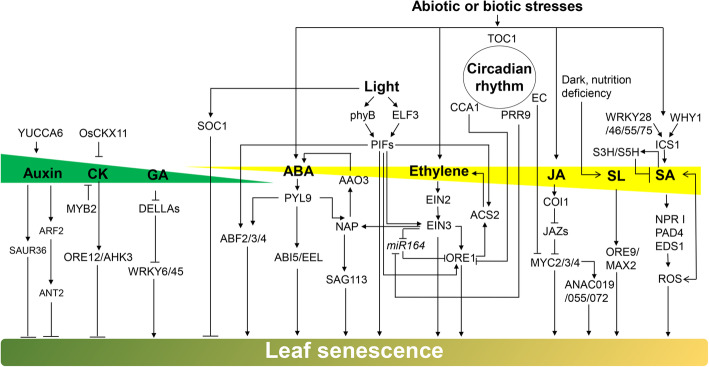
Hormonal and environmental regulation of leaf senescence. YCCA6 is responsible for the biosynthesis of auxin which inhibits leaf senescence in young leaves. The auxin responsive genes *SAUR36*, *ARF2*, and *ANT2* inhibit leaf senescence in young leaves. OsCKX11 degrades cytokinins in rice during leaf senescence. *Arabidopsis* MYB2 regulates plant senescence by affecting the cytokinin homeostasis. Activation of the cytokinin receptor AHK3 (ORE12) significantly delays leaf senescence. DELLA proteins interact with TFs WRKY45 and WRKY6 and suppress leaf senescence. ABA receptor PYL9 functions in response to ABA and actives signaling components including ABF2/3/4, ABI5/EEL and AtNAP to promote leaf senescence. *SAG113* and *AAO3* are two direct target genes of the AtNAP TF. Ethylene regulates leaf senescence through the EIN2-EIN3-miR164-ORE1 pathway. In addition, ORE1 promotes ethylene synthesis *via* activating ethylene biosynthesis gene *ACS*. JA induces leaf senescence *via* a signaling pathway involving COI1, JAZ proteins, MYC2/3/4 and NAC proteins ANAC019/055/072. SL induces leaf senescence through TFs ORE9/MAX2. WRKY family TFs WRKY28, 46, 55, 75 and the WHIRLY1 protein induce the expression of *ICS1* for SA biosynthesis and SA induces S3H and S5H which convert SA to hydroxylated SA. SA induces ROS accumulation* via* the signaling pathway NPR1-PAD4-EDS1, promoting leaf senescence. *Arabidopsis* circadian evening complex (EC) negatively regulates JA-induced leaf senescence by suppress the expression of *MYC2.* PRR9 functions as a positive regulator of leaf senescence by repressing the transcription of *miR164* and activating *ORE1* expression. CCA1 counteracts leaf senescence by directly activating *GLK2* and suppressing *ORE1* expression. ELF3 and PhyB delay leaf senescence by suppressing the transcription and protein accumulation of PIF4/PIF5. Moreover, PIF4/PIF5 directly activates expression of *EIN3*, *ABI5* and *EEL*. TF SOC1 is responsive to light and negatively regulates leaf senescence. Drought induces leaf senescence *via* activating the ABA signaling pathway. Pathogens induce the expression of WRKY TFs such as WRKY55, promote SA biosynthesis and ROS accumulation, leading to leaf senescence

Phytohormones function to integrate various developmental signals and environmental cues in navigating the senescence process through a complex network of signaling pathways, in which a fine-tuned balance between activators and repressors is maintained to ensure completion of the senescence syndrome before reaching programmed cell death (Fig. 3). It should be noted that the characterized functions of each hormone in some plant species may differ from others or plants with specific mutation background due to its complex crosstalk with other hormones.

### Cytokinins

Cytokinins are *N*^*6*^-substituted adenine derivatives essential for plant growth, development, and stress responses (Sakakibara 2006). The contents of cytokinins gradually decrease during leaf senescence (Singh et al. 1992; Gan and Amasino 1996; Zhang et al. 2021). Cytokinins delay leaf senescence in diverse plant species after exogenous application or by endogenous production through genetic approaches. The cytokinin synthesis gene *IPT* driven by a senescence-specific promoter (the *SAG12* gene promoter) significantly delays leaf senescence (Gan and Amasino 1995). Activation of the cytokinin receptor AHK3 and subsequent phosphorylation of the type B response regulator ARR2 significantly delay leaf senescence (Kim et al. 2006; Zwack and Rashotte 2013). Cytokinin response factors (CRFs) negatively regulate dark-induced leaf senescence (Zwack et al. 2016). The transcription level of *CRF6* increases when AHK3 is activated which imposes a negative regulatory effect on leaf senescence. CRF6 inhibits leaf senescence by directly or indirectly inducing the expression of downstream target genes, including cytokinin response factors *ARR6*, *ARR9* and *ARR11*, biosynthesis gene *LOG7* and transportation-related gene *ABCG14* (Zwack et al. 2016). Mutation of the TF MYB2 delays whole plant senescence by promoting cytokinin accumulation (Guo and Gan 2011). Although the function of cytokinins in leaf senescence is well known, the mechanism of cytokinin inhibition of leaf senescence remains to be elucidated. Based on the fact that increased cell-wall invertase activity triggered by cytokinins is both necessary and sufficient for the inhibition of leaf senescence, a model has been proposed in which cytokinins mediate changes in sink/source relationships that lead senescence (Zwack and Rashotte 2013). In addition, cytokinins may regulate leaf senescence through interactions with other hormones in an antagonistic or synergistic manner. For example, elevated levels of cytokinins in the rice *osckx11* mutant induce the expression of ABA degradation gene *OsABA8ox* and inhibit the expression of ABA synthesis gene *OsNCED*, thereby reducing ABA content and inhibiting leaf senescence (Zhang et al., 2021). The mechanisms that cytokinins regulate sink/source relationships and its crosstalk with other hormones remain to be further investigated for a better understanding of the role of cytokinins in leaf senescence.

### Auxin

Auxin (prominently indole-3-acetic acid, IAA) affects cell growth and plant morphogenesis. Its role in leaf senescence is complicated and somewhat controversial (Abeles et al. 1988; Lim et al. 2007). Nevertheless, it is generally appreciated that auxin is a negative regulator of leaf senescence. The endogenous IAA levels increase during leaf senescence (Quirino et al. 1999), while exogenous application of auxin inhibits the expression of SAGs (Noh and Amasino 1999), suggesting that auxin plays a negative role in leaf senescence. IAA might function at late developmental stages to prevent senescence from going too fast to ensure the completion of nutrient recycling before the cell dies. YUCCA6 encodes a flavin monooxygenase, the rate-limiting enzyme for IAA biosynthesis (Kim et al., 2007). Overexpression of YUCCA6 in *Arabidopsis* increases auxin contents and decreases the expression of *SAGs* and the accumulation of ROS, resulting in a delay of leaf senescence (Kim et al. 2011; Cha et al. 2016). Loss-of-function mutation of the auxin-responsive gene *SMALL AUXIN UP RNA 36* (*SAUR36*) delays leaf senescence, while overexpression of this gene causes earlier leaf senescence (Hou et al. 2013). Mutation in AUXIN RESPONSE FACTOR 2 (ARF2), a repressor of auxin signaling, delays leaf senescence, suggesting that auxin signaling suppresses leaf senescence (Lim et al. 2010). Meanwhile, AINTEGUMENTA (ANT), a TF belongs to the AP2/ERF family, acts downstream of ARF2 in regulating leaf senescence (Feng et al. 2016). The soybean (*Glycine max*) SENESCENCE-ASSOCIATED RECEPTOR-LIKE KINASE (SARK) and its orthologue SARK in *Arabidopsis* regulate leaf senescence through synergistic actions between auxin and ethylene (Xu et al. 2011). Auxin, as a growth hormone, probably slows down the process of leaf senescence by suppressing the actions of senescence-inducible hormones.

### Gibberellin

Gibberellins (GA) are a type of diterpene plant hormones that are biosynthesized through complex pathways and control diverse aspects of growth and development (Yamaguchi 2008). The contents of active GA in leaves gradually decrease during leaf senescence. Exogenous application of GA delays leaf senescence, while exogenous application of GA inhibitors promotes leaf senescence, suggesting that GA is a hormone that functions to slow down leaf senescence (Yu et al. 2009). Further studies have shown that GA does not directly affect leaf senescence, but it may delay leaf senescence by antagonizing ABA (Yu et al. 2009). However, several recent studies showed that GA promotes leaf senescence in some experiments. The GA signaling DELLA proteins RGL1 and RGA interact with TFs WRKY45 and WRKY6 respectively and impair their positive roles in leaf senescence (Chen et al. 2017b; Zhang et al. 2018a). The reported inconsistent functions of GA suggest that GA might indirectly regulate leaf senescence through crosstalks with other hormones in an antagonistic or synergistic manner, which is dependent on the plant species or specific mutation backgrounds.

### Ethylene

Ethylene is a gaseous plant hormone that promotes leaf senescence (Abeles et al. 1988; Jing et al. 2005). Transcriptomic studies have shown that 25% of the ethylene biosynthesis and signal transduction genes are upregulated during leaf senescence, consistent with the increase of ethylene contents in senescing leaves (Buchanan-Wollaston et al. 2005; Van der Graaff et al. 2006). Ethylene treatment promotes leaf senescence while spraying ethylene inhibitors delays leaf senescence, indicating that ethylene positively regulates leaf senescence (Abeles et al. 1988). EIN2, a central regulator of ethylene signaling, regulates *miR164*, which specifically degrades the TF ORE1, constituting an EIN2-*miR164*-ORE1 leaf senescence regulatory module (Kim et al. 2009). Further study showed that the EIN3 TF acts downstream of EIN2 and directly binds to the promoter of *miR164* and thus inhibits its expression and indirectly promotes the expression of *ORE1*, forming the EIN2-EIN3-*miR164*-ORE1 pathway regulating leaf senescence (Li et al. 2013). EIN3 was later found to directly bind to the promoters of *ORE1* and *AtNAP* to induce their transcription (Kim et al. 2014). Interestingly, over-production of ethylene in *Arabidopsis* and tomato at the early growth stage does not accelerate plants senescence, suggesting that the induction of leaf senescence by ethylene is dependent on plant age (Jing et al. 2005; Jibran et al. 2013). In addition, ethylene interacts with JA signaling in a synergistic manner in regulating leaf senescence (Tan et al. 2018; Lim et al. 2020). The Chinese flowering cabbage ERF TF BrERF72 directly activates expression of the JA biosynthesis genes *BrLOX4*, *BrAOC3*, and *BrOPR3* to induce JA production and promote leaf senescence (Tan et al. 2018). The function of ethylene in leaf senescence is well characterized in multiple plant species and ethylene has been suggested to be a downstream hormone which can directly induce leaf senescence in an age dependent mode.

### Salicylic acid

Salicylic acid (SA) is a phenolic plant hormone that plays multiple roles in plant development, biotic and abiotic stress responses (Morris et al. 2000). In *Arabidopsis*, SA contents gradually increase during leaf senescence (Breeze et al. 2011; Zhang et al. 2017b). Transgenic plants expressing the SA degrading enzyme *NahG*, the SA biosynthetic mutant *sid2*, and the signal transduction mutants *eds1*, *pad4*, and *npr1* all show delayed leaf senescence (Lim et al. 2007). SA treatment induces the expression of many *SAGs*, including the WRKY family TFs (Dong et al. 2003; Besseau et al. 2012). WRKY TFs such as WRKY75, WRKY51, WRKY28, WRKY55 and WRKY46 directly bind to the promoter region of the SA biosynthesis gene *ICS1* and promote the accumulation of SA and ROS to accelerate leaf senescence (Guo et al. 2017; Zhang et al. 2017a; Tian et al. 2020; Wang et al. 2020b). The retrograde signaling protein WHIRLY1 (WHY1) is dual-localized in chloroplasts and nuclei, regulates the expression of SA biosynthesis genes *ICS1* and *PAL*, and promotes leaf senescence *via* the SA pathway (Lin et al. 2020). The studies on SA dihydroxylases, SA 3-HYDROXYLASE (S3H) and S5H, showed that SA is involved in both the onset and the progression of leaf senescence (Zhang et al. 2013, 2017b). In addition, SA has been shown to promote leaf senescence by inducing autophagic lysosome formation (Yoshimoto et al. 2009; Xiao et al. 2010; Yin et al. 2020). It is well recognized that SA plays a direct role in both the onset and progression of leaf senescence, but most of these results are from *Arabidopsis* studies. The role of SA in leaf senescence of other plant species, especially in crop plants, remains to be investigated.

### Abscisic acid

Abscisic acid (ABA) is a plant hormone belonging to sesquiterpene, regulating plants’ response to abiotic and biotic stresses, and various developmental processes. ABA contents increase during leaf senescence and exogenous application of ABA induces leaf senescence. Expression of the NAC TF *VNI2* is induced by ABA and leaf senescence, and VNI2 functions in mediating stress-triggered leaf senescence (Yang et al. 2011). The ABA-inducible TF AtNAP promotes leaf senescence by activating its direct target genes, including the phosphatase gene *SAG113* and the ABA biosynthesis gene *AAO3* (Zhang and Gan 2012; Zhang et al. 2012; Yang et al. 2014). The TF CDF4 accelerates leaf senescence by upregulating ABA biosynthesis and repressing H_2_O_2_ scavenging (Xu et al. 2020). In rice, ABA-responsive NAC TFs OsNAC2 and ONAC054 induce the expression of ABA biosynthesis or signaling genes, which in turn enhance the expression of *SAGs* and promote leaf senescence (Liang et al. 2014; Mao et al. 2017; Sakuraba et al. 2020). ABA antagonizes cytokinins by inducing the expression of *OsCKX11* which functions to reduce cytokinin contents in senescing leaves of rice (Zhang et al. 2021). Overexpression of *OsMYB102* inhibits the expression of *SAGs*, including genes associated with ABA degradation and ABA signaling (*OsABF4*, *OsNAP*, and *OsCYP707A6*) and delays leaf senescence (Piao et al. 2019). The current data show that ABA plays a positive role in leaf senescence of different plant species including *Arabidopsis* and rice, suggesting that ABA may have a conserved role in leaf senescence and might be an ideal target for modulating the leaf senescence process.

### Jasmonic acid

Jasmonic acid (JA) is a class of lipidic plant hormones, synthesized from α-linoleic acid in the chloroplast membrane, and plays essential roles in plant development and stress responses (Wasternack 2007). JA content gradually increases during leaf senescence, and external application of JA induces leaf senescence (He et al. 2002), suggesting that JA plays a positive role in leaf senescence. *Arabidopsis* MYC2/3/4, a group of bHLH type TFs, play a central role in JA-induced leaf senescence (Zhu et al. 2015; Zhuo et al. 2020). The circadian evening complex (EC) represses JA-induced leaf senescence in *Arabidopsis* by directly binding to the promoter of *MYC2* (Zhang et al. 2018b). In addition, JA represses *CAT2* expression to increase H_2_O_2_ accumulation, thus promoting leaf senescence in a MYC2-dependent manner (Zhang et al. 2020d).

JA also regulates leaf senescence *via* crosstalks with multiple hormones. WRKY57, a repressor of JA-induced leaf senescence, interacts with JASMONATE ZIM-DOMAIN4/8 (JAZ4/8) and the AUX/IAA protein IAA29 in regulating leaf senescence through JA and IAA signaling pathways (Jiang et al. 2014). The interacting proteins WRKY53 and EPITHIOSPECIFYING SENESCENCE REGULATOR (ESR/ESP) that are antagonistically regulated by JA and SA, function in mediating negative crosstalks between pathogen resistance and senescence, which is most likely governed by the JA and SA equilibrium (Miao and Zentgraf 2007). Rice ETHYLENE RESPONSE FACTOR 101 (OsERF101) promotes the onset and progression of leaf senescence by binding to the promoters of *OsNAP* and *OsMYC2*, which activate genes involved in chlorophyll degradation and JA signaling-mediated leaf senescence (Lim et al. 2020). Wheat TaWRKY42-B promotes leaf senescence mainly by targeting a JA biosynthesis gene, *TaLOX3*, which consequently contributes to JA accumulation in dark-induced leaf senescence (Zhao et al. 2020). Apple (*Malus domestica*) BT2 protein interacts with MtJAZ protein and MtMYC2, and negatively regulates JA-triggered leaf senescence by modulating the stability of MtMYC2 and MtJAZ2 (An et al. 2020). The positive role of JA in leaf senescence has been proved in different plant species including *Arabidopsis*, rice, wheat, and apple. As a stress responsible hormone, together with ABA and SA, JA might function to integrate environmental cues into the leaf senescence process.

### Brassinolide

Brassinolide (BR) is a kind of polyhydroxy sterol, which regulates a wide range of physiological processes, including plant growth and immunity. Exogenous spray of BR accelerates leaf senescence, while the BR perception mutant *bri1* exhibits a delayed leaf senescence phenotype, indicating that BR plays a positive regulatory role during leaf senescence (Jibran et al. 2013). ATBS1-INTERACTING FACTOR 2 (AIF2), a non-DNA-binding bHLH TF, retards dark-triggered and BR-induced leaf senescence in *Arabidopsis* (Kim et al. 2020). BR signals affect the transcription and stability of AIF2 and regulate leaf senescence (Kim et al. 2020). BR as a growth-related hormone, together with auxin, CK and GA, may integrate the developmental signals to the age inducible processes of leaf senescence.

### Strigolactones

Strigolactones (SLs) are a group of terpenoid lactones synthesized from carotenoids. SLs are involved in shoot branching, root development, secondary growth, and drought tolerance (Yamada and Umehara 2015). Loss-of-function mutation of ORE9, an F-box protein identical to SL signaling protein MORE AXILALY GROWTH2 (MAX2) in *Arabidopsis*, and its orthologue OsDE3 in rice, both result in delayed leaf senescence (Woo et al. 2001; Stirnberg et al. 2002; Yan et al. 2007), suggesting that SL plays a positive role in leaf senescence. Exogenous application of G24 (a synthetic SL analog) promotes leaf senescence in the SL-deficient mutants *max1*, *max3* and *max4* of *Arabidopsis* and *d10*, *d17* and *d27* of rice (Yamada and Umehara 2015). Although GR24 treated wild-type rice do not show an early leaf senescence phenotype, the expression levels of SAGs are increased in plants, suggesting that GR24 induces SAGs during leaf senescence (Yamada et al. 2014). SL biosynthesis is induced under nitrogen or phosphate deficiency, indicating that SL serves as a signal for nutrient availability in regulating leaf senescence (Yamada et al. 2014; Yamada and Umehara 2015). The SL biosynthesis genes *MAX3* and *MAX4* are dramatically induced by dark incubation and ethylene (Ueda and Kusaba 2015). Furthermore, leaf senescence is strongly induced by the application of SL in the presence of ethylene but not by SL alone, suggesting that SL and ethylene interact synergistically in regulating leaf senescence (Ueda and Kusaba 2015). Since the amount of SL is pretty low in plants, it is tedious to quantify SL contents in different stages of leaf senescence. Future work may focus on the mechanisms of how SL integrates environmental cues into developmental and leaf senescence processes.

## Environmental regulation of leaf senescence

A range of abiotic stressors, such as drought, darkness, extreme temperature, salt and nutrition deficiency, and biotic factors, such as pathogen infection and insect attack, can accelerate the onset and/or progression of leaf senescence (Guo and Gan 2005; Lim et al. 2007; Guo and Gan 2012) (Fig. 3). However, accelerated senescence may be an active ‘escape’ strategy through which plants can reduce the canopy size in response to stresses, thereby increasing the survival rate and the chance of reproductive success (Munne-Bosch and Alegre 2004).

### Circadian rhythm

The circadian clock is closely related to aging. In plants the circadian rhythm is intertwined with leaf senescence. For example, the period of circadian rhythm is shorter (~ 22.6 h) in old leaves than that of young leaves (~ 24 h) in the same *Arabidopsis* plant (Kim et al. 2016a). TIMING OF CAB EXPRESSION 1 (TOC1), a clock oscillator, is an essential component of circadian rhythms that integrate age-related signals (Kim et al. 2016a). The *Arabidopsis* circadian EC, consisting of EARLY FLOWERING 3 (ELF3), ELF4, and MYB family TF LUX ARRHYTHMO (LUX), negatively regulates JA-induced leaf senescence by suppressing *MYC2* expression (Sanchez and Kay 2016; Zhang et al. 2018b; Woo et al. 2019). PRR9, another core component of the circadian rhythm, functions as a positive regulator of leaf senescence *via* repressing the transcription of *miR164* and consequently increasing *ORE1* expression (Kim et al. 2018). CIRCADIAN CLOCK-ASSOCIATED 1 (CCA1), a central circadian clock component, counteracts leaf senescence by directly activating *GLK2* and suppressing *ORE1* expression (Song et al. 2018), suggesting that ORE1 may be a pivotal converging node that mediates circadian rhythm-regulated leaf senescence. These findings indicate that circadian rhythm is relevant to lifespan in plants. But it remains unclear whether dysregulation of rhythm is the cause or merely a consequence of senescence.

### Light

Light is essential for plant growth and plays a critical role in regulating leaf senescence (Lim et al. 2018). Changes in light intensity, light quality and the red to far-red light ratio affect leaf senescence (Lim et al. 2018). Red light has a negative impact on leaf senescence, while far-red light plays a positive role (Thompson et al. 2000; Lim et al. 2007). PIF4 and PIF5 are crucial for darkness-induced senescence (Sakuraba et al. 2014; Song et al. 2014). ELF3 and phytochrome B (PhyB) delay leaf senescence by suppressing transcription and protein accumulation of PIF4/PIF5. Moreover, PIF4/PIF5 directly activates the expression of *EIN3*, *ABI5*, and *EEL*. In turn, PIF4/5, EIN3, ABI5 and EEL directly activate *ORE1*, thus forming multiple, coherent feed-forward loops (Sakuraba et al. 2014). PhyA and PhyB act antagonistically with WRKY6 to regulate FR-mediated leaf senescence (Sakuraba et al. 2014). FAR-RED ELONGATED HYPOCOTYL3 (FHY3) directly binds to the promoter region of *WRKY28* to repress its expression, which slows down SA biosynthesis and light-mediated leaf senescence (Tian et al. 2020).

Prolonged darkness, or complete deprivation of light over a longer time period, is one of the major inducers of leaf senescence. The transcriptome changes during dark-induced senescence largely resemble those during natural senescence (Guo and Gan 2012). Therefore, dark treatment has been widely used as a quick, simple and efficient method to synchronously induce leaf senescence, which is convenient for testing the effects of additional bioactive senescence regulators such as phytohormones, sugars, and secondary metabolites. In general, the leaf senescence phenotype caused by dark treatments is consistent with that under natural conditions. However, there are also cases where the senescence phenotype is not consistent. In this case, the senescence phenotype under natural conditions is used to determine whether a gene is positively or negatively regulating leaf senescence.

### Salt stress

Salt stress is a major cause that affects plant productivity and geographical distribution in agriculture (Zhu 2016). High salinity seriously affects the growth and development of plants, including acceleration of leaf senescence. Ionic stress, osmotic stress and secondary stresses, particularly oxidative stress, are generated under high salinity conditions to suppress normal growth and development of plants, resulting in excessive salt accumulation in leaves, thereby inducing leaf senescence and reducing plant yield (Ghanem et al. 2008; Yang and Guo 2018). When exposed to salt stress, plant cells over-accumulate ROS (Han et al. 2020). Elevated ROS levels serve as a signal that is sensed by a ROS sensor/receptor or active oxygen scavenging enzymes, including superoxide dismutase (SOD), catalase (CAT), peroxidase (POD) and others, which function to remove excessive ROS and free radicals and maintain normal growth and development (Yang and Guo 2018; Han et al. 2020). A number of TFs, such as the NAC family members, have been demonstrated to regulate salt stress-induced leaf senescence. ONAC106, a salt stress-responsive gene, negatively regulates leaf senescence in rice. The *onac106-1D* (insertion of the 35S enhancer in the promoter region of the *ONAC106* gene) mutants display delayed senescence and enhanced tolerance to salt stress (Sakuraba et al. 2015). Mutation in BILATERAL BLADE SENESCENCE 1 (OsBBS1/OsRLCK109), a rice receptor-like kinase gene, accelerates leaf senescence and attenuates salt tolerance in rice (Zeng et al. 2018). Overexpression of the salt-induced protein *salT* also delays leaf senescence in rice (Zhu et al. 2019), which might be a feedback regulation to suppress leaf senescence induced by salt stress.

### Drought stress

Drought is another critical factor affecting plant growth and survival rate. Drought stress induces a variety of responses in plants, including leaf senescence (Munne-Bosch and Alegre 2004). ABA is the major phytohormone that mediates drought-induced leaf senescence (Fujii and Zhu 2009). By activating the PP2Cs-SnRK2s-RAV1/ABF2-ORE1 signaling cascade, the ABA receptor PYL9 promotes drought resistance through limiting transpirational water loss and triggering dormancy-like responses such as senescence in old leaves and inhibiting growth in young tissues under severe drought conditions (Zhao et al. 2016). The accelerated leaf senescence in *pRD29A::PYL9* transgenic plants helps generate a greater osmotic potential gradient, allowing water to preferentially flow to developing tissues. While the acceleration of leaf senescence caused by drought positively affects plant survival and adaptability, plenty of evidence suggests that delaying leaf senescence enhances drought tolerance (Guo and Gan 2014) (See Genetic manipulation of leaf senescence for stress tolerance section). The membrane-bound TF ONAC054 is required for ABA-induced leaf senescence and is regulated at the transcriptional, post-transcriptional, and post-translational levels (Breeze 2020; Sakuraba et al. 2020). Under drought stress, the balance between growth and survival has to be well geared for plants’ fitness (Claeys and Inzé 2013), and the mechanisms underlying this are barely understood.

### Pathogen attack

In nature, plants are frequently attacked by various pathogens, leading to senescence and even death of plants. In this case, plants will initiate a series of immune defense responses to fight back. Dozens of WRKY family TFs are involved in regulating both leaf senescence and pathogen defense response, evidently through the ROS and SA pathways, both of which play important roles in leaf senescence and defense responses induced by pathogens (Zhang et al. 2020b). WRKY55 regulates the accumulation of ROS and SA by modulating the transcription of genes related to the biosynthesis of ROS and SA, thus positively regulating leaf senescence and defense against *P. syringae* (Zhang et al. 2020d). Pathogen-induced leaf senescence also involves sugar signals. Altered sensitivity to sugars and/or increased efficiency of sugar signaling in *hys1/cpr5* mutants contribute to the initiation of leaf senescence and pathogen-defense responses in *Arabidopsis* (Yoshida et al. 2002). An in-depth understanding of the regulatory mechanisms of pathogen-induced leaf senescence will help in breeding high-yield and disease-resistant crops *via* molecular breeding strategies.

### DNA-damaging stress

Plants also suffer from various types of DNA damage induced by endogenous factors and exogenous genotoxic stresses (Vijg 2000), including drought, ultraviolet light, and metabolic by-products such as ROS (Tuteja et al. 2001). Due to increased genotoxic stress and decreased DNA repair capacity, DNA damage gradually increases during normal senescence. Age-related DNA damage accumulation has been regarded as one of the main drivers of animal senescence (Vijg 2000). Recent studies revealed that a similar mechanism exists for plant senescence. As leaves age, DNA damage increases and DNA repair efficiency declines. Double-strand DNA breaks (DSBs) caused by inducible overexpression of *I-PpoI* restriction endonuclease or genotoxic chemical bleomycin accelerate leaf senescence (Li et al. 2020). Moreover, comparative analysis of transcriptomic data reveals that DSB causes gene expression changes similar to senescence. A number of the DNA repair pathway components have been shown to be involved in leaf senescence. ATAXIA TELANGIECTASIA MUTATED (ATM), a primary transducer of the DSB signal (Shiloh and Ziv 2013), is a negative regulator of leaf senescence. ATM delays senescence of *Arabidopsis* leaves by suppressing DSB-induced expression of senescence-associated TFs such as *ANAC016*, *WRKY6*, *WRKY53* and *WRKY75* *via* modulation of histone lysine methylation (Li et al. 2020). Furthermore, SUPPRESSOR OF ATM MUTANT IN FERTILITY (SATMF) plays an antagonistic role in ATM-mediated plant longevity (Zhang et al. 2020c). Nevertheless, it remains a big challenge to test whether DNA damage is a driver of senescence in plants.

## Translational research on leaf senescence for crop improvement

As summarized above, our current understanding of leaf senescence is mainly based on studies of model systems such as *Arabidopsis*. Due to the significant impact of leaf senescence on photosynthesis, nutrient remobilization, and stress responses, much effort has been made in devising strategies based on known senescence regulatory mechanisms to manipulate the initiation and progression of leaf senescence, aiming for higher yield, better quality, or improved horticultural performance in crop plants (Guo and Gan 2014; Havé et al. 2016).

### Genetic manipulation of leaf senescence for higher yield

A positive correlation between delay in leaf senescence, or the stay-green trait, and higher yield has been observed in cultivars of cereal crops such as maize (Tollenaar 1991), wheat (del Pozo et al. 2016), and sorghum (Vadez et al. 2011). Stay-green crops maintain photosynthesis capacity for a longer time after anthesis, have an extended grain-filling period, and as a consequence, higher biomass accumulation and grain yield, especially under stress conditions. During the long history of crop breeding for higher yield, genetic loci associated with stay-green traits have been selected to accumulate in most modern elite cultivars (Thomas and Ougham 2014; Kamal et al. 2019). As more and more of the stay-green loci being molecularly dissected and a large number of senescence regulators identified from model plant systems, during the past two decades, a significant amount of work has been done in genetic manipulation of crop plants to delay leaf senescence (Guo and Gan 2014; Havé et al. 2016; Woo et al. 2019).

As an example of successful senescence manipulation through genetic approaches, the senescence-specific enhancement of cytokinin accumulation system has been widely utilized to effectively delay leaf senescence in a large number of plant species (Guo and Gan 2014). This autoregulatory system is designed based on the senescence-inhibiting effect of cytokinins with the cytokinin biosynthetic *Isopentenyl transferase* (*IPT*) gene driven by the senescence-specific *SAG12* promoter (Gan and Amasino 1995). Significant delay of leaf senescence has been observed in transgenic plants harboring p*SAG12::IPT* or *IPT* driven by other senescence-inducible promoters, with higher yield often achieved in crop plants (Guo and Gan 2014; Kant et al. 2015; Décima Oneto et al. 2016; Joshi et al. 2019) (Table 1).

**Table 1:** Effects of delayed leaf senescence on yield of genetically engineered crop plants

Plant species	Strategies for delaying senescence	Organs harvested	Yield increase (%)	Growth conditions	Note	References
Cassava	p*SAG12::IPT*	Storage root	−23.33	Field	Increased drought tolerance	Zhang et al. 2010
Canola	p*AtMYB32xs::IPT*	Grain	16–23 or 7–16	Field, rainfed or irrigated	Elevated oleic acid content	Kant et al. 2015
Maize	p*SARK::IPT*	Grain	47–66	Green house water deficit	No effect when well-watered	Décima Oneto et al. 2016
Maize	*HaHB11* overexpression	Grain	28	Field		Raineri et al. 2019
Maize	*nac7* RNAi	Grain	2.2–3.5	Field		Zhang et al. 2019
Peanut	p*SARK::IPT*	Seed	51	Field, water-limiting	No yield increase when well-watered	Qin et al. 2011
Rice	p*SARK::IPT*	Grain	144	Green house, water-stressed	No yield increase when well-watered	Peleg et al. 2011
Rice	OsNAP RNAi	Grain	6.3–10.3	Field	Reduced grain protein and mineral micronutrient	Liang et al. 2014
Rice	*OsGATA12* overexpression	Grain	30	Green house, high density	Reduced yield at low density	Lu et al. 2017
Rice	*ONAC096* T-DNA insertion	Grain	16	Field		Kang et al. 2019
Rice	*OsCKX11 Crispr/Cas9*	Grain	7.5	Field	Increased grain number	Zhang et al. 2021
Rice	Introgression of japonica *OsSGR* allele	Grain	10.6–12.7	Field		Shin et al. 2020
Tomato	*SlORE1S02* RNAi	Fruit	43–95^a^	Green house	Increased soluble solid content	Lira et al. 2017
Tomato	*SlNAP2* RNAi	Fruit	19^a^	Growth chamber	Increased soluble solid content	Ma et al. 2018
Wheat	*TaNAM* RNAi	Grain	Not affected	Field	Reduced grain protein, zinc content	Uauy et al. 2006
Wheat	EMS mutagenesis of GPC-A1 and GPC-D1	Grain	Not affected	Field	Reduced grain protein content	Avni et al. 2014
Wheat	*TaNAC-S* overexpression	Grain	Not affected	Green house	Increased straw N content	Zhao et al. 2015
Wheat	*NAM* RNAi	Grain	Not affected	Growth room	Increased stem fructans	Borrill et al. 2015
Wheat	p*SAG12::IPT*	Grain	Not affected	Green house		Sýkorová et al. 2008
Wheat	p*AtMYB32xs::IPT*	Grain	40–67	Field, water stressed	Slight increase when well-watered	Joshi et al. 2019

^a^Estimated based on bar graphs when numbers are not provided in the article

Other than the IPT-based senescence-inhibiting approach, major senescence regulators, mostly senescence-regulating TFs, NAC family TFs in particular, have been used in genetic manipulation of leaf senescence (Liang et al. 2014; Borrill et al. 2015; Lira et al. 2017; Raineri et al. 2019; Zhang et al. 2019). NAP and ORE1 are NAC family TFs that were initially identified in *Arabidopsis* as master regulators of leaf senescence (Guo and Gan 2006; Kim et al. 2009). Homolog genes in a number of plant species of AtNAP and AtORE1 have been characterized to have similar functions in regulating leaf senescence (Guo and Gan 2014; Liang et al. 2014; Lira et al. 2017; Ma et al. 2018). Changes in expression of these TFs in crop plants lead to a significant delay in leaf senescence and in many cases, yield increase (Lu et al. 2017; Ma et al. 2018; Kang et al. 2019; Raineri et al. 2019).

It is promising to see that yield increase *via* delaying leaf senescence has been achieved in diverse crop species including rice (Peleg et al. 2011; Liang et al. 2014; Lu et al. 2017; Kang et al. 2019; Zhang et al. 2021), maize (Décima Oneto et al. 2016; Raineri et al. 2019; Zhang et al. 2019), wheat (Joshi et al. 2019), tomato (Lira et al. 2017; Ma et al. 2018), canola (Kant et al. 2015), and peanut (Qin et al. 2011). Delayed leaf senescence however, does not always result in increased yield. A 23% reduction in yield of storage root was observed when the pSAG12::IPT strategy was applied on cassava, accompanied by a significant delay in leaf senescence (Zhang et al. 2010). In several other cases, a significant delay in leaf senescence did not affect yield (Uauy et al. 2006; Sýkorová et al. 2008; Avni et al. 2014; Borrill et al. 2015; Zhao et al. 2015) (Table 1).

The senescence process of leaves could impact crop yield though two opposite directions: delayed leaf senescence on one side could increase yield due to prolonged photosynthesis and an extended grain-filling period while on the other hand, delay in leaf senescence could compromise nutrient remobilization from senescing leaves to organs to be harvested, which may negatively affect yield (Wu et al. 2012b; Gregersen et al. 2013; Distelfeld et al. 2014). In a number of cases, delayed senescence was associated with reduced grain protein content (Uauy et al. 2006; Avni et al. 2014; Liang et al. 2014), indicating delayed nitrogen remobilization from the source leaves. Also, the yield potential of cereals is believed to be the result of the synergistic interaction between source activity and sink capacity (Lv et al. 2020). Sink strength, not source activity which is affected by leaf senescence, has been suggested to be the limiting factor in yield formation, at least for some crops such as wheat (Borrás et al. 2004). Since source-sink interaction and nutrient remobilization during senescence seem to be dependent on plant species, harvesting organs, and growth conditions (Havé et al. 2016; Kamal et al. 2019), studies targeting specific crop species are necessary to facilitate utilization of our current understanding of leaf senescence and nutrient remobilization for yield and quality improvement in agriculture.

### Genetic manipulation of leaf senescence for stress tolerance

It is almost always the case that plants with delayed leaf senescence show higher tolerance to environmental stresses, including drought, salinity, low nitrogen, flooding, cold and heat (Guo and Gan 2014). As described in the previous section, senescence can be triggered by a variety of biotic as well as abiotic stresses (Ali et al. 2018; Woo et al. 2019), and significant similarity in gene expression is found between senescence and plants’ response to different stress conditions (Guo and Gan 2012). Under stress conditions, source activity instead of sink strength might become the limiting factor for crop performance. Therefore, it’s not surprising to see that the benefit of delaying leaf senescence is more significant when plants are facing unfavorable environmental conditions. Leaf senescence was significantly delayed in wheat plants expressing the *IPT* gene driven by a modified *AtMYB32xs* promoter. Transgenic wheat plants were grown in the field with irrigation (well-watered) or water-stressed with rainout shelters. While the delayed senescence transgenic plants showed 40–67% increase in grain yield compared with wild-type plants under water-stressed conditions, yield increase of the stay-green plants was not significant under the well-watered treatments (Joshi et al. 2019). Similarly, rice (Peleg et al. 2011) and peanut (Qin et al. 2011) plants harboring p*SARK::IPT* showed significant yield increase under water-limiting conditions but no yield increase was observed when these plants were well-watered. In another study, overexpression of the zinc finger TF gene *OsGATA12* led to a 30% increase in grain yield in rice plants grown in high density. When the *OsGATA12* expressing plants were grown in low density, grain yield was decreased instead (Lu et al. 2017). With global climate changes and the requirement of reducing nitrate fertilizers, crop plants are imposed with increasingly harsher growth conditions while higher production is required to feed the world’s increasing population. It is critical to develop stress-tolerant plants that keep their photosynthetic capacity and maintain productivity under high temperature, water-limited conditions, and reduced fertilization.

### Genetic manipulation of leaf senescence for better horticultural performance

In addition to yield/biomass increase as in cereals, delayed leaf senescence could also improve the quality and performance of horticultural crops. The p*SAG12::IPT* system has been used in manipulating senescence in vegetable crops including lettuce, broccoli, cauliflower, and bok choy with a significant extension of the shelf life after harvest (reviewed in Guo and Gan 2014). Green leaf color is a key trait for green-leaf vegetables such as Chinese cabbage (*Brassica rapa ssp. pekinensis*) and pakchoi (*Brassica campestris L. ssp. chinensis*). Stay-green mutants of these crops have been generated *via* mutagenesis targeting the key Chl-degradation genes NYE1/SGR1 (Wang et al. 2018, 2020a). Creeping bentgrass (*Agrostis stolonifera*), a cool-season specialty turfgrass primarily used for golf courses, is sensitive to environmental stresses. Using the p*SAG12::IPT* strategy, a series of studies by the Huang group obtained creeping bentgrass with enhanced tolerance to drought and heat stresses (Merewitz et al. 2016; Xu et al. 2016b), and increased root viability (Xu et al. 2010; Merewitz et al. 2011). Interestingly, delay of leaf senescence in fruit harvesting crops was shown to improve the quality of fleshy fruits. In two independent studies, tomato leaf senescence was manipulated by suppressing the expression of NAC family TFs *SlORE1S02* (Lira et al. 2017) and *SiNAP* (Ma et al. 2018) *via* RNAi. As a result of significantly delayed leaf senescence, a significant increase in fruit yield was achieved in both studies. In addition, increased soluble solid contents were observed in tomato fruits from the transgenic plants (Lira et al. 2017; Ma et al. 2018). It appears that for fruit crop like tomatoes, prolonged photosynthesis capacity does not prevent nutrient translocation from source to sink tissues. Higher contents in sugars such as fructose, sucrose, glucose were observed in fruits of delayed-senescence tomato, which increased sweetness of the fruits (Lira et al. 2017; Ma et al. 2018). Recent progress in apple leaf senescence could potentially provide more information on leaf senescence and fruit quality in fleshy fruit production (Hu et al. 2019a; Han et al. 2020).

## Conclusions

Impressive progress has been achieved in understanding leaf senescence through forward/reverse genetic strategies and omics-based technologies, etc. However, we still know too little about how leaf senescence is regulated.

Although each phytohormone’s function and signaling, especially that of ethylene, ABA, JA, and SA, are well-acknowledged in leaf senescence, the crosstalks between these phytohormones and how they cooperate with each other to manipulate the senescence process remain to be elucidated. Meanwhile, how the developmental signals and environmental cues are integrated into the hormonal signaling pathways and how these signals are finely controlled through TFs, chromatin modifiers, RNA modifiers, and protein modifiers to regulate gene expression and protein turnover during leaf senescence, remain to be big challenges in this field.

For translational research, the source-sink relationship and nutrient remobilization during the grain-filling period of cereals need to be addressed at the molecular level. Studies on model plants may not be able to provide a precise prediction for specific crop species. With the publication of the genomes of many plant species, functional analysis of *SAG*s in different species will deepen our understanding of the relationship between senescence and yield. With the application of gene-editing technologies such as CRISPR/Cas9, we anticipate that more and more genome modified stay-green crops will be developed and commercialized in the near future.

## Data Availability

Not applicable.

## Abbreviations

ABA: Abscisic acid
ABI5: ABA INSENSITIVE 5
ABS3: ABNORMAL SHOOT 3
AIF2: ATBS1-INTERACTING FACTOR 2
ANT: AINTEGUMENTA
ARF2: AUXIN RESPONSE FACTOR 2
AS: Alternative splicing
ATG: AUTOPHAGY
ATI1: ATG8-INTERACTING PROTEIN1
ATL31: ARABIDOPSIS TOXICOS EN LEVADURA 31
ATM: ATAXIA TELANGIECTASIA MUTATED
BBS1: BILATERAL BLADE SENESCENCE 1
BCM1: BALANCE of CHLOROPHYLL METABOLISM 1
BRs: Brassinosteroids
BSP: Bark storage proteins
CAT: Catalase
CCA1: CIRCADIAN CLOCK-ASSOCIATED 1
CCEs: Chlorophyll catabolic enzymes
CCGs: Chlorophyll catabolic genes
CCVs: CV-containing vesicles
Chl: Chlorophyll
CKs: Cytokinins
CRFs: Cytokinin response factors
CRN1: Co-regulated with NYE1
CV: CHLOROPLAST VESICULATION
DDM1: DECREASED DNA METHYLATION 1
DFCC: Dioxobilin-type (type-II) FCC
DNCC: Dioxobilin-type (type-II) NCC
DRD1: DEFECTIVE IN RNA-DIRECTED DNA METHYLATION 1
DSBs: Double-strand DNA breaks
DUBs: Deubiquitination enzymes
EC: Evening complex
EEL: ENHANCED EM LEVEL
EIN2: ETHYLENE INSENSITIVE 2
ELF3: EARLY FLOWERING 3
ERF4: ETHYLENE RESPONSE FACTOR4
ESP: EPITHIOSPECIFIER PROTEIN
ESR: EPITHIOSPECIFYING SENESCENCE REGULATOR
FCC: Fluorescent chlorophyll catabolite
FHY3: FAR-RED ELONGATED HYPOCOTYL 3
GA: Gibberellic acid
H3K27me3: Trimethylation of lysine 27 on histone H3 protein
H3K4me3: Imethylation of lysine 4 on histone H3 protein
H3K9ac: Cetylation of lysine 9 on histone H3 protein
HDA9: HISTONE DEACETYLASE 9
IPT: Isopentenyl transferase
JA: Jasmonic acid
JAZ: JASMONATE ZIM-DOMAIN
LUX: LUX ARRHYTHMO
MATE: Multidrug and toxic compound extrusion
MAX2: MORE AXILALY GROWTH 2
mFCC: Modified FCC
NAP: NAC-LIKE, ACTIVATED BY AP3/PI
NCC: Non-fluorescent chlorophyll catabolite
NOL: NYC1-like
NORE1: NOT ORESARA 1
NPX1: NUCLEAR PROTEIN X 1
NYC1: NON-YELLOW COLORING 1
NYEs: NON-YELLOWINGs
OEM: Outer-envelope-membrane
ORE1: ORESARA 1
pFCCs: Primary fluorescent chlorophyll catabolites
PhyB: Phytochrome B
PIF: PHYTOCHROME-INTERACTING FACTOR
POD: Peroxidase
PTMs: Post-translational modifications
RCB: Rubisco-containing bodies
RCC: Red chlorophyll catabolite
ROS: Reactive oxygen species
S3H: SA 3-HYDROXYLASE
SA: Salicylic acid
SAGs: Senescence-associated genes
SAM: Shoot apical meristem
SARK: SENESCENCE- ASSOCIATED RECEPTOR-LIKE KINASE
SATMF: SUPPRESSOR OF ATM MUTANT IN FERTILITY
SAUL1: SENESCENCE-ASSOCIATED E3 UBIQUITIN LIGASE 1
SAUR36: SMALL AUXIN UP RNA 36
SGRL: SGR-LIKE
SGRs: STAY-GREENs
SLs: Strigolactones
smRNAs: Small RNAs
SOD: Superoxide dismutase
SSTG: Small starch granule-like structures
SUVH2: Suppressor of variegation 3–9-homologous 2
tasiRNAs: Trans-acting small interference RNAs
TCP: TEOSINTE BRANCHED/CYCLOIDEA
TFs: Transcription factors
TOC: Translocon outer membrane complex
TOC1: TIMING OF CAB EXPRESSION 1
UBPs: Ubiquitin-specific proteases
UPS: Ubiquitin-26S proteasome system

## References

[CR1] Abeles FB, Dunn LJ, Morgens P, Callahan A, Dinterman RE, Schmidt J (1988). Induction of 33kD and 60kD peroxidases during ethylene-induced senescence of cucumber cotyledons. Plant Physiol.

[CR2] Agüera E, De la Haba P (2018). Leaf senescence in response to elevated atmospheric CO_2_ concentration and low nitrogen supply. Biol Plantarum.

[CR3] Ahmad S, Guo Y (2019). Signal transduction in leaf senescence: progress and perspective. Plants.

[CR4] Ali A, Gao X, Guo Y (2018). Initiation, progression, and genetic manipulation of leaf senescence. Methods Mol Biol.

[CR5] An JP, Wang XF, Zhang XW, You CX, Hao YJ (2020). Apple BT2 protein negatively regulates jasmonic acid-triggered leaf senescence by modulating the stability of MYC2 and JAZ2. Plant Cell Environ.

[CR6] Aoyama S, Huarancca Reyes T, Guglielminetti L, Lu Y, Morita Y, Sato T, Yamaguchi J (2014). Ubiquitin ligase ATL31 functions in leaf senescence in response to the balance between atmospheric CO2 and nitrogen availability in Arabidopsis. Plant Cell Physiol.

[CR7] Archacki R, Yatusevich R, Buszewicz D, Krzyczmonik K, Patryn J, Iwanicka-Nowicka R, Biecek P, Wilczynski B, Koblowska M, Jerzmanowski A, Swiezewski S (2017). Arabidopsis SWI/SNF chromatin remodeling complex binds both promoters and terminators to regulate gene expression. Nucleic Acids Res.

[CR8] Armstead I, Donnison I, Aubry S, Harper J, Hortensteiner S, James C, Mani J, Moffet M, Ougham H, Roberts L, Thomas A, Weeden N, Thomas H, King I (2007). Cross-species identification of Mendel’s/locus. Science.

[CR9] Aubry S, Fankhauser N, Ovinnikov S, Pruzinska A, Stirnemann M, Zienkiewicz K, Herrfurth C, Feussner I, Hortensteiner S (2020). Pheophorbide a may regulate jasmonate signaling during dark-induced senescence. Plant Physiol.

[CR10] Avni R, Zhao R, Pearce S, Jun Y, Uauy C, Tabbita F, Fahima T, Slade A, Dubcovsky J, Distelfeld A (2014). Functional characterization of GPC-1 genes in hexaploid wheat. Planta.

[CR11] Ay N, Irmler K, Fischer A, Uhlemann R, Reuter G, Humbeck K (2009). Epigenetic programming via histone methylation at WRKY53 controls leaf senescence in Arabidopsis thaliana. Plant J.

[CR12] Bai F, Corll J, Shodja DN, Davenport R, Feng G, Mudunkothge J, Brigolin CJ, Martin F, Spielbauer G, Tseung CW, Siebert AE, Barbazuk WB, Lal S, Settles AM (2019). RNA binding motif protein 48 is required for U12 splicing and maize endosperm differentiation. Plant Cell.

[CR13] Barbazuk WB, Fu Y, McGinnis KM (2008). Genome-wide analyses of alternative splicing in plants: opportunities and challenges. Genome Res.

[CR14] Barry CS, McQuinn RP, Chung MY, Besuden A, Giovannoni JJ (2008). Amino acid substitutions in homologs of the STAY-GREEN protein are responsible for the green-flesh and chlorophyll retainer mutations of tomato and pepper. Plant Physiol.

[CR15] Besseau S, Li J, Palva ET (2012). WRKY54 and WRKY70 co-operate as negative regulators of leaf senescence in Arabidopsis thaliana. J Exp Bot.

[CR16] Book AJ, Smalle J, Lee K-H, Yang P, Walker JM, Casper S, Holmes JH, Russo LA, Buzzinotti ZW, Jenik PD, Vierstra RD (2009). The RPN5 subunit of the 26s proteasome is essential for gametogenesis, sporophyte development, and complex assembly in Arabidopsis. Plant Cell.

[CR17] Borrás L, Slafer GA, Otegui ME (2004). Seed dry weight response to source-sink manipulations in wheat, maize and soybean: a quantitative reappraisal. Field Crops Res.

[CR18] Borrill P, Fahy B, Smith AM, Uauy C (2015). Wheat grain filling is limited by grain filling capacity rather than the duration of flag leaf photosynthesis: a case study using NAM RNAi plants. PLoS One.

[CR19] Breeze E (2020). Make, modify, move: multilayered regulation of ONAC054 during ABA-induced leaf senescence. Plant Cell.

[CR20] Breeze E, Harrison E, McHattie S, Hughes L, Hickman R, Hill C, Kiddle S, Kim YS, Penfold CA, Jenkins D, Zhang C, Morris K, Jenner C, Jackson S, Thomas B, Tabrett A, Legaie R, Moore JD, Wild DL, Ott S, Rand D, Beynon J, Denby K, Mead A, Buchanan-Wollaston V (2011). High-resolution temporal profiling of transcripts during Arabidopsis leaf senescence reveals a distinct chronology of processes and regulation. Plant Cell.

[CR21] Brusslan JA, Bonora G, Rus-Canterbury AM, Tariq F, Jaroszewicz A, Pellegrini M (2015). A genome-wide chronological study of gene expression and two histone modifications, H3K4me3 and H3K9ac, during developmental leaf senescence. Plant Physiol.

[CR22] Buchanan-Wollaston V, Page T, Harrison E, Breeze E, Lim PO, Nam HG, Lin JF, Wu SH, Swidzinski J, Ishizaki K, Leaver CJ (2005). Comparative transcriptome analysis reveals significant differences in gene expression and signalling pathways between developmental and dark/starvation-induced senescence in Arabidopsis. Plant J.

[CR23] Buet A, Costa ML, Martinez DE, Guiamet JJ (2019). Chloroplast protein degradation in senescing leaves: proteases and lytic compartments. Front Plant Sci.

[CR24] Carrion CA, Lorenza Costa M, Martinez DE, Mohr C, Humbeck K, Guiamet JJ (2013). In vivo inhibition of cysteine proteases provides evidence for the involvement of ‘senescence-associated vacuoles’ in chloroplast protein degradation during dark-induced senescence of tobacco leaves. J Exp Bot.

[CR25] Cha JY, Kim MR, Jung IJ, Kang SB, Park HJ, Kim MG, Yun DJ, Kim WY (2016). The thiol reductase activity of YUCCA6 mediates delayed leaf senescence by regulating genes involved in auxin redistribution. Front Plant Sci.

[CR26] Chen J, Zhu X, Ren J, Qiu K, Li Z, Xie Z, Gao J, Zhou X, Kuai B (2017). Suppressor of overexpression of CO 1 negatively regulates dark-induced leaf degreening and senescence by directly repressing pheophytinase and other senescence-associated genes in Arabidopsis. Plant Physiol.

[CR27] Chen L, Xiang S, Chen Y, Li D, Yu D (2017). Arabidopsis WRKY45 interacts with the DELLA protein RGL1 to positively regulate age-triggered leaf senescence. Mol Plant.

[CR28] Chen M (2014). Chlorophyll modifications and their spectral extension in oxygenic photosynthesis. Annu Rev Biochem.

[CR29] Chen X, Lu L, Mayer KS, Scalf M, Qian S, Lomax A, Smith LM, Zhong X (2016). POWERDRESS interacts with HISTONE DEACETYLASE 9 to promote aging in Arabidopsis. Elife.

[CR30] Chi W, Sun X, Zhang L (2013). Intracellular signaling from plastid to nucleus. Annu Rev Plant Biol.

[CR31] Cho EJ, Choi SH, Kim JH, Kim JE, Lee MH, Chung BY, Woo HR, Kim J-H (2016). A mutation in plant-specific SWI2/SNF2-like chromatin-remodeling proteins, DRD1 and DDM1, delays leaf senescence in Arabidopsis thaliana. PLoS One.

[CR32] Christ B, Hortensteiner S (2014). Mechanism and significance of chlorophyll breakdown. J Plant Growth Regul.

[CR33] Christ B, Schelbert S, Aubry S, Suessenbacher I, Mueller T, Kraeutler B, Hoertensteiner S (2012). MES16, a member of the methylesterase protein family, specifically demethylates fluorescent chlorophyll catabolites during chlorophyll breakdown in Arabidopsis. Plant Physiol.

[CR34] Christ B, Suessenbacher I, Moser S, Bichsel N, Egert A, Mueller T, Kraeutler B, Hoertensteiner S (2013). Cytochrome P450 CYP89A9 is involved in the formation of major chlorophyll catabolites during leaf senescence in Arabidopsis. Plant Cell.

[CR35] Chung DW, Pružinská A, Hörtensteiner S, Ort DR (2006). The role of pheophorbide oxygenase expression and activity in the canola green seed problem. Plant Physiol.

[CR36] Claeys H, Inzé D. The Agony of Choice: How Plants Balance Growth and Survival under Water-Limiting Conditions. Plant Physiology. 2013;162(4):1768-79. 10.1104/pp.113.220921 PMC3729759 23766368

[CR37] Cooke JE, Weih M (2005). Nitrogen storage and seasonal nitrogen cycling in Populus: bridging molecular physiology and ecophysiology. New Phytol.

[CR38] Cui X, Lu F, Li Y, Xue Y, Kang Y, Zhang S, Qiu Q, Cui X, Zheng S, Liu B, Xu X, Cao X (2013). Ubiquitin-specific proteases UBP12 and UBP13 act in circadian clock and photoperiodic flowering regulation in Arabidopsis. Plant Physiol.

[CR39] Décima Oneto C, Otegui ME, Baroli I, Beznec A, Faccio P, Bossio E, Blumwald E, Lewi D (2016). Water deficit stress tolerance in maize conferred by expression of an isopentenyltransferase (IPT) gene driven by a stress- and maturation-induced promoter. J Biotechnol.

[CR40] del Pozo A, Yáñez A, Matus IA, Tapia G, Castillo D, Sanchez-Jardón L, Araus JL (2016). Physiological traits associated with wheat yield potential and performance under water-stress in a Mediterranean environment. Front Plant Sci.

[CR41] Delmas F, Sankaranarayanan S, Deb S, Widdup E, Bournonville C, Bollier N, Northey JGB, McCourt P, Samuel MA (2013). ABI3 controls embryo degreening through Mendel’s I locus. Proc Natl Acad Sci U S A.

[CR42] Diaz C, Lemaitre T, Christ A, Azzopardi M, Kato Y, Sato F, Morot-Gaudry J-F, Le Dily F, Masclaux-Daubresse C (2008). Nitrogen recycling and remobilization are differentially controlled by leaf senescence and development stage in Arabidopsis under low nitrogen nutrition. Plant Physiol.

[CR43] Distelfeld A, Avni R, Fischer AM (2014). Senescence, nutrient remobilization, and yield in wheat and barley. J Exp Bot.

[CR44] Dong J, Chen C, Chen Z (2003). Expression profiles of the Arabidopsis WRKY gene superfamily during plant defense response. Plant Mol Biol.

[CR45] Efroni I, Han S-K, Kim HJ, Wu M-F, Steiner E, Birnbaum KD, Hong JC, Eshed Y, Wagner D (2013). Regulation of leaf maturation by chromatin-mediated modulation of cytokinin responses. Dev Cell.

[CR46] Feng GP, Xu Q, Wang ZY, Zhuoma QJ (2016). AINTEGUMENTA negatively regulates age-dependent leaf senescence downstream of AUXIN RESPONSE FACTOR 2 in Arabidopsis thaliana. Plant Biotechnol.

[CR47] Frank S, Hollmann J, Mulisch M, Matros A, Carrion CC, Mock HP, Hensel G, Krupinska K (2019). Barley cysteine protease PAP14 plays a role in degradation of chloroplast proteins. J Exp Bot.

[CR48] Fujii H, Zhu JK (2009). Arabidopsis mutant deficient in 3 abscisic acid-activated protein kinasesreveals critical roles in growth, reproduction, and stress. Proc Natl Acad Sci U S A.

[CR49] Gan S, Amasino RM (1995). Inhibition of leaf senescence by autoregulated production of cytokinin. Science.

[CR50] Gan S, Amasino RM (1996). Cytokinins in plant senescence: from spray and pray to clone and play. BioEssays.

[CR51] Gan S, Amasino RM (1997). Making sense of senescence (molecular genetic regulation and manipulation of leaf senescence). Plant Physiol.

[CR52] Gao S, Gao J, Zhu X, Song Y, Li Z, Ren G, Zhou X, Kuai B (2016). ABF2, ABF3, and ABF4 promote ABA-mediated chlorophyll degradation and leaf senescence by transcriptional activation of chlorophyll catabolic genes and senescence-associated genes in Arabidopsis. Mol Plant.

[CR53] Gault CM, Martin F, Mei W, Bai F, Black JB, Barbazuk WB, Settles AM (2017). Aberrant splicing in maize rough endosperm3 reveals a conserved role for U12 splicing in eukaryotic multicellular development. Proc Natl Acad Sci U S A.

[CR54] Ghanem ME, Albacete A, Martinez-Andujar C, Acosta M, Romero-Aranda R, Dodd IC, Lutts S, Perez-Alfocea F (2008). Hormonal changes during salinity-induced leaf senescence in tomato (Solanum lycopersicum L.). J Exp Bot.

[CR55] Goossens J, Mertens J, Goossens A (2017). Role and functioning of bHLH transcription factors in jasmonate signalling. J Exp Bot.

[CR56] Gregersen PL, Culetic A, Boschian L, Krupinska K (2013). Plant senescence and crop productivity. Plant Mol Biol.

[CR57] Guo P, Li Z, Huang P, Li B, Fang S, Chu J, Guo H (2017). A tripartite amplification loop involving the transcription factor WRKY75, salicylic acid, and reactive oxygen species accelerates leaf senescence. Plant Cell.

[CR58] Guo Y (2013). Towards systems biological understanding of leaf senescence. Plant Mol Biol.

[CR59] Guo Y, Gan S (2005). Leaf senescence: signals, execution, and regulation. Curr Top Dev Biol.

[CR60] Guo Y, Gan S (2006). AtNAP, a NAC family transcription factor, has an important role in leaf senescence. Plant J.

[CR61] Guo Y, Gan S (2011). AtMYB2 regulates whole plant senescence by inhibiting cytokinin-mediated branching at late stages of development in Arabidopsis. Plant Physiol.

[CR62] Guo Y, Gan SS (2014). Translational researches on leaf senescence for enhancing plant productivity and quality. J Exp Bot.

[CR63] Guo Y, Gan S-S (2012). Convergence and divergence in gene expression profiles induced by leaf senescence and 27 senescence-promoting hormonal, pathological and environmental stress treatments. Plant Cell Environ.

[CR64] Hackett JB, Shi X, Kobylarz AT, Lucas MK, Wessendorf RL, Hines KM, Bentolila S, Hanson MR, Lu Y (2017). An organelle RNA recognition motif protein is required for photosystem II subunit psbF transcript editing. Plant Physiol.

[CR65] Han D, Du M, Zhou Z, Wang S, Li T, Han J, Xu T, Yang G (2020). Overexpression of a Malus baccata NAC transcription factor gene MbNAC25 increases cold and salinity tolerance in Arabidopsis. Int J Mol Sci.

[CR66] Hauenstein M, Christ B, Das A, Aubry S, Hortensteiner S (2016). A role for TIC55 as a hydroxylase of phyllobilins, the products of chlorophyll breakdown during plant senescence. Plant Cell.

[CR67] Havé M, Balliau T, Cottyn-Boitte B, Dérond E, Cueff G, Soulay F, Lornac A, Reichman P, Dissmeyer N, Avice J-C, Gallois P, Rajjou L, Zivy M, Masclaux-Daubresse C (2018). Increases in activity of proteasome and papain-like cysteine protease in Arabidopsis autophagy mutants: back-up compensatory effect or cell-death promoting effect?. J Exp Bot.

[CR68] Havé M, Marmagne A, Chardon F, Masclaux-Daubresse C (2016). Nitrogen remobilization during leaf senescence: lessons from Arabidopsis to crops. J Exp Bot.

[CR69] He L, Wu W, Zinta G, Yang L, Wang D, Liu R, Zhang H, Zheng Z, Huang H, Zhang Q, Zhu J-K (2018). A naturally occurring epiallele associates with leaf senescence and local climate adaptation in Arabidopsis accessions. Nat Commun.

[CR70] He Y, Fukushige H, Hildebrand DF, Gan S (2002). Evidence supporting a role of jasmonic acid in Arabidopsis leaf senescence. Plant Physiol.

[CR71] Hickman R, Hill C, Penfold CA, Breeze E, Bowden L, Moore JD, Zhang P, Jackson A, Cooke E, Bewicke-Copley F, Mead A, Beynon J, Wild DL, Denby KJ, Ott S, Buchanan-Wollaston V (2013). A local regulatory network around three NAC transcription factors in stress responses and senescence in Arabidopsis leaves. Plant J.

[CR72] Hillwig MS, Contento AL, Meyer A, Ebany D, Bassham DC, Macintosh GC (2011). RNS2, a conserved member of the RNase T2 family, is necessary for ribosomal RNA decay in plants. Proc Natl Acad Sci U S A.

[CR73] Hinckley WE, Keymanesh K, Cordova JA, Brusslan JA (2019). The HAC1 histone acetyltransferase promotes leaf senescence and regulates the expression of ERF022. Plant Direct.

[CR74] Hoertensteiner S (2009). Stay-green regulates chlorophyll and chlorophyll-binding protein degradation during senescence. Trends Plant Sci.

[CR75] Hoertensteiner S (2013). Update on the biochemistry of chlorophyll breakdown. Plant Mol Biol.

[CR76] Horie Y, Ito H, Kusaba M, Tanaka R, Tanaka A (2009). Participation of chlorophyll b reductase in the initial step of the degradation of light-harvesting chlorophyll a/b-protein complexes in Arabidopsis. J Biol Chem.

[CR77] Hortensteiner S, Feller U (2002). Nitrogen metabolism and remobilization during senescence. J Exp Bot.

[CR78] Hou K, Wu W, Gan SS (2013). SAUR36, a small auxin up RNA gene, is involved in the promotion of leaf senescence in Arabidopsis. Plant Physiol.

[CR79] Hu DG, Yu JQ, Han PL, Xie XB, Sun CH, Zhang QY, Wang JH, Hao YJ (2019). The regulatory module MdPUB29-MdbHLH3 connects ethylene biosynthesis with fruit quality in apple. New Phytol.

[CR80] Hu Y, Lu Y, Zhao Y, Zhou D-X (2019). Histone acetylation dynamics integrates metabolic activity to regulate plant response to stress. Front Plant Sci.

[CR81] Huang CK, Lo PC, Huang LF, Wu SJ, Yeh CH, Lu CA (2015). A single-repeat MYB transcription repressor, MYBH, participates in regulation of leaf senescence in Arabidopsis. Plant Mol Biol.

[CR82] Huang D, Lan W, Li D, Deng B, Lin W, Ren Y, Miao Y (2018). WHIRLY1 occupancy affects histone lysine modification and WRKY53 transcription in Arabidopsis developmental manner. Front Plant Sci.

[CR83] Ishida H, Yoshimoto K, Izumi M, Reisen D, Yano Y, Makino A, Ohsumi Y, Hanson MR, Mae T (2008). Mobilization of rubisco and stroma-localized fluorescent proteins of chloroplasts to the vacuole by an ATG gene-dependent autophagic process. Plant Physiol.

[CR84] Izumi M, Ishida H, Nakamura S, Hidema J (2017). Entire photodamaged chloroplasts are transported to the central vacuole by autophagy. Plant Cell.

[CR85] Jaradat MR, Feurtado JA, Huang D, Lu Y, Cutler AJ (2013). Multiple roles of the transcription factor AtMYBR1/AtMYB44 in ABA signaling, stress responses, and leaf senescence. BMC Plant Biol.

[CR86] Jia M, Liu X, Xue H, Wu Y, Shi L, Wang R, Chen Y, Xu N, Zhao J, Shao J, Qi Y, An L, Sheen J, Yu F (2019). Noncanonical ATG8-ABS3 interaction controls senescence in plants. Nat Plants.

[CR87] Jiang H, Li M, Liang N, Yan H, Wei Y, Xu X, Liu J, Xu Z, Chen F, Wu G (2007). Molecular cloning and function analysis of the stay green gene in rice. Plant J.

[CR88] Jiang Y, Liang G, Yang S, Yu D (2014). Arabidopsis WRKY57 functions as a node of convergence for jasmonic acid- and auxin-mediated signaling in jasmonic acid-induced leaf senescence. Plant Cell.

[CR89] Jibran R, Hunter DA, Dijkwel PP (2013). Hormonal regulation of leaf senescence through integration of developmental and stress signals. Plant Mol Biol.

[CR90] Jing HC, Schippers JH, Hille J, Dijkwel PP (2005). Ethylene-induced leaf senescence depends on age-related changes and OLD genes in Arabidopsis. J Exp Bot.

[CR91] Joshi S, Choukimath A, Isenegger D, Panozzo J, Spangenberg G, Kant S (2019). Improved wheat growth and yield by delayed leaf senescence using developmentally regulated expression of a cytokinin biosynthesis gene. Front Plant Sci.

[CR92] Kamal NM, Gorafi YS, Abdelrahman M, Abdellatef E, Tsujimoto H (2019). Stay-green trait: a prospective approach for yield potential, and drought and heat stress adaptation in globally important cereals. Int J Mol Sci.

[CR93] Kang K, Shim Y, Gi E, An G, Paek NC (2019). Mutation of ONAC096 enhances grain yield by increasing panicle number and delaying leaf senescence during grain filling in rice. Int J Mol Sci.

[CR94] Kant S, Burch D, Badenhorst P, Palanisamy R, Mason J, Spangenberg G (2015). Regulated expression of a cytokinin biosynthesis gene IPT delays leaf senescence and improves yield under rainfed and irrigated conditions in canola (Brassica napus L.). PLoS One.

[CR95] Kato Y, Murakami S, Yamamoto Y, Chatani H, Kondo Y, Nakano T, Yokota A, Sato F (2004). The DNA-binding protease, CND41, and the degradation of ribulose-1,5-bisphosphate carboxylase/oxygenase in senescent leaves of tobacco. Planta.

[CR96] Kato Y, Yamamoto Y, Murakami S, Sato F (2005). Post-translational regulation of CND41 protease activity in senescent tobacco leaves. Planta.

[CR97] Keskitalo J, Bergquist G, Gardestrom P, Jansson S (2005). A cellular timetable of autumn senescence. Plant Physiol.

[CR98] Kikuchi Y, Nakamura S, Woodson JD, Ishida H, Ling Q, Hidema J, Jarvis RP, Hagihara S, Izumi M (2020). Chloroplast autophagy and ubiquitination combine to manage oxidative damage and starvation responses. Plant Physiol.

[CR99] Kim JI, Sharkhuu A, Jin JB, Li P, Jeong JC, Baek D, Lee SY, Blakeslee JJ, Murphy AS, Bohnert HJ, Hasegawa PM, Yun D-J, Bressan RA. yucca6, a dominant mutation in Arabidopsis, affects auxin accumulation and auxin-related phenotypes. Plant Physiol. 2007;145(3):722-35. 10.1104/pp.107.104935 PMC2048792 17885085

[CR100] Kim H, Kim Y, Yeom M, Lim J, Nam HG (2016). Age-associated circadian period changes in Arabidopsis leaves. J Exp Bot.

[CR101] Kim HJ, Hong SH, Kim YW, Lee IH, Jun JH, Phee BK, Rupak T, Jeong H, Lee Y, Hong BS, Nam HG, Woo HR, Lim PO (2014). Gene regulatory cascade of senescence-associated NAC transcription factors activated by ETHYLENE-INSENSITIVE2-mediated leaf senescence signalling in Arabidopsis. J Exp Bot.

[CR102] Kim HJ, Nam HG, Lim PO (2016). Regulatory network of NAC transcription factors in leaf senescence. Curr Opin Plant Biol.

[CR103] Kim HJ, Park JH, Kim J, Kim JJ, Hong S, Kim J, Kim JH, Woo HR, Hyeon C, Lim PO, Nam HG, Hwang D (2018). Time-evolving genetic networks reveal a NAC troika that negatively regulates leaf senescence in Arabidopsis. Proc Natl Acad Sci U S A.

[CR104] Kim HJ, Ryu H, Hong SH, Woo HR, Lim PO, Lee IC, Sheen J, Nam HG, Hwang I (2006). Cytokinin-mediated control of leaf longevity by AHK3 through phosphorylation of ARR2 in Arabidopsis. Proc Natl Acad Sci U S A.

[CR105] Kim JH, Woo HR, Kim J, Lim PO, Lee IC, Choi SH, Hwang D, Nam HG (2009). Trifurcate feed-forward regulation of age-dependent cell death involving miR164 in Arabidopsis. Science.

[CR106] Kim JI, Murphy AS, Baek D, Lee SW, Yun DJ, Bressan RA, Narasimhan ML (2011). YUCCA6 over-expression demonstrates auxin function in delaying leaf senescence in Arabidopsis thaliana. J Exp Bot.

[CR107] Kim Y, Park SU, Shin DM, Pham G, Jeong YS, Kim SH (2020). ATBS1-INTERACTING FACTOR 2 negatively regulates dark- and brassinosteroid-induced leaf senescence through interactions with INDUCER OF CBF EXPRESSION 1. J Exp Bot.

[CR108] Koyama T, Nii H, Mitsuda N, Ohta M, Kitajima S, Ohme-Takagi M, Sato F (2013). A regulatory cascade involving class II ETHYLENE RESPONSE FACTOR transcriptional repressors operates in the progression of leaf senescence. Plant Physiol.

[CR109] Kuai B, Chen J, Hortensteiner S (2018). The biochemistry and molecular biology of chlorophyll breakdown. J Exp Bot.

[CR110] Kusaba M, Ito H, Morita R, Iida S, Sato Y, Fujimoto M, Kawasaki S, Tanaka R, Hirochika H, Nishimura M, Tanaka A (2007). Rice NON-YELLOW COLORING1 is involved in light-harvesting complex II and grana degradation during leaf senescence. Plant Cell.

[CR111] Kusaba M, Maoka T, Morita R, Takaichi S (2009). A novel carotenoid derivative, lutein 3-acetate, accumulates in senescent leaves of Rice. Plant Cell Physiol.

[CR112] Lan W, Miao Y (2019). New aspects of HECT-E3 ligases in cell senescence and cell death of plants. Plants.

[CR113] Li C, Gu L, Gao L (2016). Concerted genomic targeting of H3K27 demethylase REF6 and chromatin-remodeling ATPase BRM in Arabidopsis. Nat Genet.

[CR114] Li F, Chung T, Vierstra RD (2014). AUTOPHAGY-RELATED11 plays a critical role in general autophagy- and senescence-induced mitophagy in Arabidopsis. Plant Cell.

[CR115] Li Z, Peng J, Wen X, Guo H (2013). Ethylene-insensitive3 is a senescence-associated gene that accelerates age-dependent leaf senescence by directly repressing miR164 transcription in Arabidopsis. Plant Cell.

[CR116] Li Z, Woo HR, Guo H (2018). Genetic redundancy of senescence-associated transcription factors in Arabidopsis. J Exp Bot.

[CR117] Li Z, Wu S, Chen J, Wang X, Gao J, Ren G, Kuai B (2017). NYEs/SGRs-mediated chlorophyll degradation is critical for detoxification during seed maturation in Arabidopsis. Plant J.

[CR118] Li Z, Zhang Y, Zou D, Zhao Y, Wang HL, Zhang Y, Xia X, Luo J, Guo H, Zhang Z (2020). LSD 3.0: a comprehensive resource for the leaf senescence research community. Nucleic Acids Res.

[CR119] Liang C, Wang Y, Zhu Y, Tang J, Hu B, Liu L, Ou S, Wu H, Sun X, Chu J, Chu C (2014). OsNAP connects abscisic acid and leaf senescence by fine-tuning abscisic acid biosynthesis and directly targeting senescence-associated genes in rice. Proc Natl Acad Sci U S A.

[CR120] Lim C, Kang K, Shim Y, Sakuraba Y, An G, Paek NC (2020). Rice ETHYLENE RESPONSE FACTOR 101 promotes leaf senescence through Jasmonic acid-mediated regulation of OsNAP and OsMYC2. Front Plant Sci.

[CR121] Lim J, Park JH, Jung S, Hwang D, Nam HG, Hong S (2018). Antagonistic roles of PhyA and PhyB in far-red light-dependent leaf senescence in Arabidopsis thaliana. Plant Cell Physiol.

[CR122] Lim PO, Kim HJ, Nam HG (2007). Leaf senescence. Annu Rev Plant Biol.

[CR123] Lim PO, Lee IC, Kim J, Kim HJ, Ryu JS, Woo HR, Nam HG (2010). Auxin response factor 2 (ARF2) plays a major role in regulating auxin-mediated leaf longevity. J Exp Bot.

[CR124] Lin W, Zhang H, Huang D, Schenke D, Cai D, Wu B, Miao Y (2020). Dual-localized WHIRLY1 affects salicylic acid biosynthesis via coordination of ISOCHORISMATE SYNTHASE1, PHENYLALANINE AMMONIA LYASE1 and S-ADENOSYL-L-METHIONINE-DEPENDENT METHYLTRANSFERASE1. Plant Physiol.

[CR125] Lin YL, Sung SC, Tsai HL, Yu TT, Radjacommare R, Usharani R, Fatimababy AS, Lin HY, Wang YY, Fu H (2011). The defective proteasome but not substrate recognition function is responsible for the null phenotypes of the Arabidopsis proteasome subunit RPN10. Plant Cell.

[CR126] Ling Q, Huang W, Baldwin A, Jarvis P (2012). Chloroplast biogenesis is regulated by direct action of the ubiquitin-proteasome system. Science.

[CR127] Ling QH, Broad W, Trosch R, Topel M, Sert TD, Lymperopoulos P, Baldwin A, Jarvis RP (2019). Ubiquitin-dependent chloroplast-associated protein degradation in plants. Science.

[CR128] Lira BS, Gramegna G, Trench BA, Alves FRR, Silva EM, Silva GFF, Thirumalaikumar VP, Lupi ACD, Demarco D, Purgatto E, Nogueira FTS, Balazadeh S, Freschi L, Rossi M (2017). Manipulation of a senescence-associated gene improves fleshy fruit yield. Plant Physiol.

[CR129] Liu P, Zhang S, Zhou B, Luo X, Zhou XF, Cai B, Jin YH, Niu D, Lin J, Cao X, Jin JB (2019). The histone H3K4 demethylase JMJ16 represses leaf senescence in Arabidopsis. Plant Cell.

[CR130] Lu G, Casaretto JA, Ying S, Mahmood K, Liu F, Bi YM, Rothstein SJ (2017). Overexpression of OsGATA12 regulates chlorophyll content, delays plant senescence and improves rice yield under high density planting. Plant Mol Biol.

[CR131] Lv X, Zhang Y, Zhang Y, Fan S, Kong L (2020). Source-sink modifications affect leaf senescence and grain mass in wheat as revealed by proteomic analysis. BMC Plant Biol.

[CR132] Lyons R, Iwase A, Gänsewig T, Sherstnev A, Duc C, Barton GJ, Hanada K, Higuchi-Takeuchi M, Matsui M, Sugimoto K (2013). The RNA-binding protein FPA regulates flg22-triggered defense responses and transcription factor activity by alternative polyadenylation. Sci Rep.

[CR133] Ma X, Zhang Y, Turečková V, Xue G-P, Fernie AR, Mueller-Roeber B, Balazadeh S (2018). The NAC transcription factor SlNAP2 regulates leaf senescence and fruit yield in tomato. Plant Physiol.

[CR134] Mao C, Lu S, Lv B, Zhang B, Shen J, He J, Luo L, Xi D, Chen X, Ming F (2017). A rice NAC transcription factor promotes leaf senescence via ABA biosynthesis. Plant Physiol.

[CR135] Marshall RS, Li F, Gemperline DC, Book AJ, Vierstra RD (2015). Autophagic degradation of the 26S proteasome is mediated by the dual ATG8/ubiquitin receptor RPN10 in Arabidopsis. Mol Cell.

[CR136] Martinez DE, Costa ML, Gomez FM, Otegui MS, Guiamet JJ (2008). ‘Senescence-associated vacuoles’ are involved in the degradation of chloroplast proteins in tobacco leaves. Plant J.

[CR137] Masclaux-Daubresse C, Clément G, Anne P, Routaboul J-M, Guiboileau A, Soulay F, Shirasu K, Yoshimoto K (2014). Stitching together the multiple dimensions of autophagy using metabolomics and transcriptomics reveals impacts on metabolism, development, and plant responses to the environment in Arabidopsis. Plant Cell.

[CR138] Meguro M, Ito H, Takabayashi A, Tanaka R, Tanaka A (2011). Identification of the 7-hydroxymethyl chlorophyll a reductase of the chlorophyll cycle in Arabidopsis. Plant Cell.

[CR139] Merewitz E, Xu Y, Huang B (2016). Differentially expressed genes associated with improved drought tolerance in creeping Bentgrass overexpressing a gene for cytokinin biosynthesis. PLoS One.

[CR140] Merewitz EB, Gianfagna T, Huang B (2011). Protein accumulation in leaves and roots associated with improved drought tolerance in creeping bentgrass expressing an ipt gene for cytokinin synthesis. J Exp Bot.

[CR141] Miao Y, Laun T, Zimmermann P, Zentgraf U (2004). Targets of the WRKY53 transcription factor and its role during leaf senescence in Arabidopsis. Plant Mol Biol.

[CR142] Miao Y, Zentgraf U (2007). The antagonist function of Arabidopsis WRKY53 and ESR/ESP in leaf senescence is modulated by the jasmonic and salicylic acid equilibrium. Plant Cell.

[CR143] Miao Y, Zentgraf U (2010). A HECT E3 ubiquitin ligase negatively regulates Arabidopsis leaf senescence through degradation of the transcription factor WRKY53. Plant J.

[CR144] Michaeli S, Honig A, Levanony H, Peled-Zehavi H, Galili G (2014). Arabidopsis ATG8-INTERACTING PROTEIN1 is involved in autophagy-dependent vesicular trafficking of plastid proteins to the vacuole. Plant Cell.

[CR145] Minina EA, Moschou PN, Vetukuri RR, Sanchez-Vera V, Cardoso C, Liu Q, Elander PH, Dalman K, Beganovic M, Lindberg Yilmaz J, Marmon S, Shabala L, Suarez MF, Ljung K, Novák O, Shabala S, Stymne S, Hofius D, Bozhkov PV (2018). Transcriptional stimulation of rate-limiting components of the autophagic pathway improves plant fitness. J Exp Bot.

[CR146] Morris K, MacKerness SA, Page T, John CF, Murphy AM, Carr JP, Buchanan-Wollaston V (2000). Salicylic acid has a role in regulating gene expression during leaf senescence. Plant J.

[CR147] Mueller T, Ulrich M, Ongania K-H, Kraeutler B (2007). Colorless tetrapyrrolic chlorophyll catabolites found in ripening fruit are effective antioxidants. Angew Chem (Int Ed Engl).

[CR148] Munne-Bosch S, Alegre L (2004). Die and let live: leaf senescence contributes to plant survival under drought stress. Funct Plant Biol.

[CR149] Mur LAJ, Aubry S, Mondhe M, Kingston-Smith A, Gallagher J, Timms-Taravella E, James C, Papp I, Hoertensteiner S, Thomas H, Ougham H (2010). Accumulation of chlorophyll catabolites photosensitizes the hypersensitive response elicited by Pseudomonas syringae in Arabidopsis. New Phytol.

[CR150] Nakamura S, Hidema J, Sakamoto W, Ishida H, Izumi M (2018). Selective elimination of membrane-damaged chloroplasts via microautophagy. Plant Physiol.

[CR151] Noh YS, Amasino RM (1999). Identification of a promoter region responsible for the senescence-specific expression of SAG12. Plant Mol Biol.

[CR152] Oberhuber M, Berghold J, Kraeutler B (2008). Chlorophyll breakdown by a biomimetic route. Angew Chem Int Ed Engl.

[CR153] Ono K, Kimura M, Matsuura H, Tanaka A, Ito H (2019). Jasmonate production through chlorophyll a degradation by stay-green in Arabidopsis thaliana. J Plant Physiol.

[CR154] Otegui MS (2018). Vacuolar degradation of chloroplast components: autophagy and beyond. J Exp Bot.

[CR155] Otegui MS, Noh YS, Martinez DE, Vila Petroff MG, Andrew Staehelin L, Amasino RM, Guiamet JJ (2005). Senescence-associated vacuoles with intense proteolytic activity develop in leaves of Arabidopsis and soybean. Plant J.

[CR156] Pak C, Van Doorn WG (2004). Delay of Iris flower senescence by protease inhibitors. New Phytol.

[CR157] Park SH, Jeong JS, Seo JS, Park BS, Chua NH (2019). Arabidopsis ubiquitin-specific proteases UBP12 and UBP13 shape ORE1 levels during leaf senescence induced by nitrogen deficiency. New Phytol.

[CR158] Park SY, Yu JW, Park JS, Li J, Yoo SC, Lee NY, Lee SK, Jeong SW, Seo HS, Koh HJ, Jeon JS, Park YI, Paek NC (2007). The senescence-induced staygreen protein regulates chlorophyll degradation. Plant Cell.

[CR159] Peleg Z, Reguera M, Tumimbang E, Walia H, Blumwald E (2011). Cytokinin-mediated source/sink modifications improve drought tolerance and increase grain yield in rice under water-stress. Plant Biotechnol J.

[CR160] Penfold CA, Buchanan-Wollaston V (2014). Modelling transcriptional networks in leaf senescence. J Exp Bot.

[CR161] Piao W, Kim SH, Lee BD, An G, Sakuraba Y, Paek NC (2019). Rice transcription factor OsMYB102 delays leaf senescence by down-regulating abscisic acid accumulation and signaling. J Exp Bot.

[CR162] Poovaiah BW, Leopold AC (1973). Deferral of leaf senescence with calcium. Plant Physiol.

[CR163] Poret M, Chandrasekar B, van der Hoorn RAL, Avice J-C (2016). Characterization of senescence-associated protease activities involved in the efficient protein remobilization during leaf senescence of winter oilseed rape. Plant Sci.

[CR164] Pruzinska A, Anders I, Aubry S, Schenk N, Tapernoux-Luethi E, Mueller T, Kraeutler B, Hoertensteiner S (2007). In vivo participation of red chlorophyll catabolite reductase in chlorophyll breakdown. Plant Cell.

[CR165] Pruzinska A, Tanner G, Anders I, Roca M, Hortensteiner S (2003). Chlorophyll breakdown: Pheophorbide a oxygenase is a Rieske-type iron-sulfur protein, encoded by the accelerated cell death 1 gene. Proc Natl Acad Sci U S A.

[CR166] Pruzinska A, Tanner G, Aubry S, Anders I, Moser S, Muller T, Ongania KH, Krautler B, Youn JY, Liljegren SJ, Hortensteiner S (2005). Chlorophyll breakdown in senescent Arabidopsis leaves. Characterization of chlorophyll catabolites and of chlorophyll catabolic enzymes involved in the degreening reaction. Plant Physiol.

[CR167] Qi T, Wang J, Huang H, Liu B, Gao H, Liu Y, Song S, Xie D (2015). Regulation of jasmonate-induced leaf senescence by antagonism between bHLH subgroup IIIe and IIId factors in Arabidopsis. Plant Cell.

[CR168] Qin H, Gu Q, Zhang J, Sun L, Kuppu S, Zhang Y, Burow M, Payton P, Blumwald E, Zhang H (2011). Regulated expression of an isopentenyltransferase gene (IPT) in peanut significantly improves drought tolerance and increases yield under field conditions. Plant Cell Physiol.

[CR169] Qin J, Ma X, Yi Z, Tang Z, Meng Y (2015). A transcriptome-wide study on the microRNA- and the Argonaute 1-enriched small RNA-mediated regulatory networks involved in plant leaf senescence. Plant Biol.

[CR170] Qiu K, Li Z, Yang Z, Chen J, Wu S, Zhu X, Gao S, Gao J, Ren G, Kuai B, Zhou X (2015). EIN3 and ORE1 accelerate degreening during ethylene-mediated leaf senescence by directly activating chlorophyll catabolic genes in Arabidopsis. PLoS Genet.

[CR171] Quirino BF, Normanly J, Amasino RM (1999). Diverse range of gene activity during Arabidopsis thaliana leaf senescence includes pathogen-independent induction of defense-related genes. Plant Mol Biol.

[CR172] Raineri J, Campi M, Chan RL, Otegui ME (2019). Maize expressing the sunflower transcription factor HaHB11 has improved productivity in controlled and field conditions. Plant Sci.

[CR173] Ren G, An K, Liao Y, Zhou X, Cao Y, Zhao H, Ge X, Kuai B (2007). Identification of a novel chloroplast protein AtNYE1 regulating chlorophyll degradation during leaf senescence in Arabidopsis. Plant Physiol.

[CR174] Ren GD, Zhou Q, Wu SX, Zhang YF, Zhang LG, Huang JR, Sun ZF, Kuai BK (2010). Reverse genetic identification of CRN1 and its distinctive role in chlorophyll degradation in Arabidopsis. J Integr Plant Biol.

[CR175] Ren Y, Li Y, Jiang Y, Wu B, Miao Y (2017). Phosphorylation of WHIRLY1 by CIPK14 shifts its localization and dual functions in Arabidopsis. Mol Plant.

[CR176] Ren Y, Wang W, Lan W, Schenke D, Cai D, Miao Y. MicroRNA840 accelerates leaf senescence by targeting the overlapping 3’UTRs of PPR and WHIRLY3 in Arabidopsis thaliana. bioRxiv. 2020. 10.1101/2020.10.23.353052. 10.1111/tpj.15559 34724261

[CR177] Riester L, Koester-Hofmann S, Doll J, Berendzen KW, Zentgraf U (2019). Impact of alternatively Polyadenylated isoforms of ETHYLENE RESPONSE FACTOR4 with activator and repressor function on senescence in Arabidopsis thaliana L. Genes.

[CR178] Rivero RM, Kojima M, Gepstein A, Sakakibara H, Mittler R, Gepstein S, Blumwald E (2007). Delayed leaf senescence induces extreme drought tolerance in a flowering plant. Proc Natl Acad Sci U S A.

[CR179] Robatzek S, Somssich IE (2001). A new member of the Arabidopsis WRKY transcription factor family, AtWRKY6, is associated with both senescence- and defence-related processes. Plant J.

[CR180] Roberts IN, Caputo C, Criado MV, Funk C (2012). Senescence-associated proteases in plants. Physiol Plant.

[CR181] Sade N, Del Mar Rubio-Wilhelmi M, Umnajkitikorn K, Blumwald E (2018). Stress-induced senescence and plant tolerance to abiotic stress. J Exp Bot.

[CR182] Sakakibara H (2006). Cytokinins: activity, biosynthesis, and translocation. Annu Rev Plant Biol.

[CR183] Sakuraba Y, Jeong J, Kang MY, Kim J, Paek NC, Choi G (2014). Phytochrome-interacting transcription factors PIF4 and PIF5 induce leaf senescence in Arabidopsis. Nat Commun.

[CR184] Sakuraba Y, Kim D, Han SH, Kim SH, Piao W, Yanagisawa S, An G, Paek NC (2020). Multilayered regulation of membrane-bound ONAC054 is essential for abscisic acid-induced leaf senescence in rice. Plant Cell.

[CR185] Sakuraba Y, Piao W, Lim JH, Han SH, Kim YS, An G, Paek NC (2015). Rice ONAC106 inhibits leaf senescence and increases salt tolerance and tiller angle. Plant Cell Physiol.

[CR186] Sakuraba Y, Schelbert S, Park SY, Han SH, Lee BD, Andres CB, Kessler F, Hortensteiner S, Paek NC (2012). STAY-GREEN and chlorophyll catabolic enzymes interact at light-harvesting complex II for chlorophyll detoxification during leaf senescence in Arabidopsis. Plant Cell.

[CR187] Sanchez SE, Kay SA (2016). The plant circadian clock: from a simple timekeeper to a complex developmental manager. Cold Spring Harb Perspect Biol.

[CR188] Sanchez SE, Rugnone ML, Kay SA (2020). Light perception: a matter of time. Mol Plant.

[CR189] Sato T, Shimoda Y, Matsuda K, Tanaka A, Ito H (2018). Mg-dechelation of chlorophyll a by Stay-Green activates chlorophyll b degradation through expressing non-yellow coloring 1 in Arabidopsis thaliana. J Plant Physiol.

[CR190] Sato Y, Morita R, Katsuma S, Nishimura M, Tanaka A, Kusaba M (2009). Two short-chain dehydrogenase/reductases, NON-YELLOW COLORING 1 and NYC1-LIKE, are required for chlorophyll b and light-harvesting complex II degradation during senescence in rice. Plant J.

[CR191] Schelbert S, Aubry S, Burla B, Agne B, Kessler F, Krupinska K, Hoertensteiner S (2009). Pheophytin pheophorbide hydrolase (pheophytinase) is involved in chlorophyll breakdown during leaf senescence in Arabidopsis. Plant Cell.

[CR192] Schommer C, Palatnik JF, Aggarwal P, Chételat A, Cubas P, Farmer EE, Nath U, Weigel D (2008). Control of jasmonate biosynthesis and senescence by miR319 targets. PLoS Biol.

[CR193] Schreiber A, Peter M (2014). Substrate recognition in selective autophagy and the ubiquitin–proteasome system. Biochim Biophys Acta (BBA) - Mol Cell Res.

[CR194] Shibata M, Oikawa K, Yoshimoto K, Kondo M, Mano S, Yamada K, Hayashi M, Sakamoto W, Ohsumi Y, Nishimura M (2013). Highly oxidized peroxisomes are selectively degraded via autophagy in Arabidopsis. Plant Cell.

[CR195] Shiloh Y, Ziv Y (2013). The ATM protein kinase: regulating the cellular response to genotoxic stress, and more. Nat Rev Mol Cell Biol.

[CR196] Shimoda Y, Ito H, Tanaka A (2016). Arabidopsis STAY-GREEN, Mendel’s green cotyledon gene, encodes magnesium-dechelatase. Plant Cell.

[CR197] Shin D, Lee S, Kim T-H, Lee J-H, Park J, Lee J, Lee JY, Cho L-H, Choi JY, Lee W, Park J-H, Lee D-W, Ito H, Kim DH, Tanaka A, Cho J-H, Song Y-C, Hwang D, Purugganan MD, Jeon J-S, An G, Nam HG (2020). Natural variations at the Stay-Green gene promoter control lifespan and yield in rice cultivars. Nat Commun.

[CR198] Shu K, Yang W (2017). E3 ubiquitin ligases: ubiquitous actors in plant development and abiotic stress responses. Plant Cell Physiol.

[CR199] Singh S, Letham DS, Palni LMS (1992). Cytokinin biochemistry in relation to leaf senescence. VII. Endogenous cytokinin levels and exogenous applications of cytokinins in relation to sequential leaf senescence of tobacco. Physiol Plant.

[CR200] Slotte T, Huang HR, Holm K, Ceplitis A, St. Onge K, Chen J, Lagercrantz U, Lascoux M (2009). Splicing variation at a FLOWERING LOCUS C homeolog is associated with flowering time variation in the tetraploid Capsella bursa-pastoris. Genetics.

[CR201] Song Y, Jiang Y, Kuai B, Li L (2018). CIRCADIAN CLOCK-ASSOCIATED 1 inhibits leaf senescence in Arabidopsis. Front Plant Sci.

[CR202] Song Y, Yang C, Gao S, Zhang W, Li L, Kuai B (2014). Age-triggered and dark-induced leaf senescence require the bHLH transcription factors PIF3, 4, and 5. Mol Plant.

[CR203] Stirnberg P, van De Sande K, Leyser HM (2002). MAX1 and MAX2 control shoot lateral branching in Arabidopsis. Development.

[CR204] Sun YK, Gutmann B, Yap A, Kindgren P, Small I (2018). Editing of chloroplast rps14 by PPR editing factor EMB2261 is essential for Arabidopsis development. Front Plant Sci.

[CR205] Sýkorová B, Kurešová G, Daskalova S, Trčková M, Hoyerová K, Raimanová I, Motyka V, Trávníčková A, Elliott MC, Kamínek M (2008). Senescence-induced ectopic expression of the A. tumefaciens ipt gene in wheat delays leaf senescence, increases cytokinin content, nitrate influx, and nitrate reductase activity, but does not affect grain yield. J Exp Bot.

[CR206] Tan XL, Fan ZQ, Shan W, Yin XR, Kuang JF, Lu WJ, Chen JY (2018). Association of BrERF72 with methyl jasmonate-induced leaf senescence of Chinese flowering cabbage through activating JA biosynthesis-related genes. Hortic Res.

[CR207] Thatcher SR, Burd S, Wright C, Lers A, Green PJ (2015). Differential expression of miRNAs and their target genes in senescing leaves and siliques: insights from deep sequencing of small RNAs and cleaved target RNAs. Plant Cell Environ.

[CR208] Thomas H, Ougham H (2014). The stay-green trait. J Exp Bot.

[CR209] Thompson J, Taylor C, Wang TW (2000). Altered membrane lipase expression delays leaf senescence. Biochem Soc Trans.

[CR210] Tian F, Yu J, Zhang Y, Xie Y, Wu B, Miao Y (2019). MORF9 functions in plastid RNA editing with tissue specificity. Int J Mol Sci.

[CR211] Tian T, Ma L, Liu Y, Xu D, Chen Q, Li G (2020). Arabidopsis FAR-RED ELONGATED HYPOCOTYL3 integrates age and light signals to negatively regulate leaf senescence. Plant Cell.

[CR212] Tollenaar M (1991). Physiological basis of genetic improvement of maize hybrids in Ontario from 1959 to 1988. Crop Sci.

[CR213] Tuteja N, Singh MB, Misra MK, Bhalla PL, Tuteja R (2001). Molecular mechanisms of DNA damage and repair: progress in plants. Crit Rev Biochem Mol Biol.

[CR214] Uauy C, Distelfeld A, Fahima T, Blechl A, Dubcovsky J (2006). A NAC gene regulating senescence improves grain protein, zinc, and iron content in wheat. Science.

[CR215] Ueda H, Kusaba M (2015). Strigolactone regulates leaf senescence in concert with ethylene in Arabidopsis. Plant Physiol.

[CR216] Ueda M, Seki M (2020). Histone modifications form epigenetic regulatory networks to regulate abiotic stress response. Plant Physiol.

[CR217] Vadez V, Deshpande SP, Kholova J, Hammer GL, Borrell AK, Talwar HS, Hash CT (2011). Stay-green quantitative trait loci’s effects on water extraction, transpiration efficiency and seed yield depend on recipient parent background. Funct Plant Biol.

[CR218] Van der Graaff E, Schwacke R, Schneider A, Desimone M, Flugge UI, Kunze R (2006). Transcription analysis of Arabidopsis membrane transporters and hormone pathways during developmental and induced leaf senescence. Plant Physiol.

[CR219] Van Wijk KJ, Merchant SS (2015). Protein maturation and proteolysis in plant plastids, mitochondria, and peroxisomes. Annual review of plant biology.

[CR220] Velasco-Arroyo B, Diaz-Mendoza M, Gandullo J, Gonzalez-Melendi P, Santamaria ME, Dominguez-Figueroa JD, Hensel G, Martinez M, Kumlehn J, Diaz I (2016). HvPap-1 C1A protease actively participates in barley proteolysis mediated by abiotic stresses. J Exp Bot.

[CR221] Vierstra RD (2009). The ubiquitin–26S proteasome system at the nexus of plant biology. Nat Rev Mol Cell Biol.

[CR222] Vijg J (2000). Somatic mutations and aging: a re-evaluation. Mutat Res.

[CR223] Vogelmann K, Drechsel G, Bergler J, Subert C, Philippar K, Soll J, Engelmann JC, Engelsdorf T, Voll LM, Hoth S (2012). Early senescence and cell death in Arabidopsis saul1 mutants involve the PAD4-dependent salicylic acid pathway. Plant Physiol.

[CR224] Wagner R, Aigner H, Pruzinska A, Jankanpaa HJ, Jansson S, Funk C (2011). Fitness analyses of Arabidopsis thaliana mutants depleted of FtsH metalloproteases and characterization of three FtsH6 deletion mutants exposed to high light stress, senescence and chilling. New Phytol.

[CR225] Wang H, Schippers JHM (2019). The role and regulation of autophagy and the proteasome during aging and senescence in plants. Genes.

[CR226] Wang N, Liu Z, Zhang Y, Li C, Feng H (2018). Identification and fine mapping of a stay-green gene (Brnye1) in pakchoi (Brassica campestris L. ssp. chinensis). Theor Appl Genet.

[CR227] Wang N, Zhang Y, Huang S, Liu Z, Li C, Feng H (2020). Defect in Brnym1, a magnesium-dechelatase protein, causes a stay-green phenotype in an EMS-mutagenized Chinese cabbage (Brassica campestris L. ssp. pekinensis) line. Hortic Res.

[CR228] Wang SH, Blumwald E (2014). Stress-induced chloroplast degradation in Arabidopsis is regulated via a process independent of autophagy and senescence-associated vacuoles. Plant Cell.

[CR229] Wang X, Gao J, Gao S, Song Y, Yang Z, Kuai B (2019). The H3K27me3 demethylase REF6 promotes leaf senescence through directly activating major senescence regulatory and functional genes in Arabidopsis. PLoS Genet.

[CR230] Wang Y, Cui X, Yang B, Xu S, Wei X, Zhao P, Niu F, Sun M, Wang C, Cheng H, Jiang YQ (2020). WRKY55 transcription factor positively regulates leaf senescence and the defense response by modulating the transcription of genes implicated in the biosynthesis of reactive oxygen species and salicylic acid in Arabidopsis. Development.

[CR231] Wang Y, Yu BJ, Zhao JP, Guo JB, Li Y, Han SJ, Huang L, Du YM, Hong YG, Tang DZ, Liu YL (2013). Autophagy contributes to leaf starch degradation. Plant Cell.

[CR232] Wasternack C (2007). Jasmonates: an update on biosynthesis, signal transduction and action in plant stress response, growth and development. Ann Bot.

[CR233] Willems P, Horne A, Van Parys T, Goormachtig S, De Smet I, Botzki A, Van Breusegem F, Gevaert K (2019). The plant PTM viewer, a central resource for exploring plant protein modifications. Plant J.

[CR234] Wingler A, Marès M, Pourtau N (2004). Spatial patterns and metabolic regulation of photosynthetic parameters during leaf senescence. New Phytol.

[CR235] Woo HR, Chung KM, Park JH, Oh SA, Ahn T, Hong SH, Jang SK, Nam HG (2001). ORE9, an F-box protein that regulates leaf senescence in Arabidopsis. Plant Cell.

[CR236] Woo HR, Goh CH, Park JH, dela Serve BT, Kim JH, Park YL, Nam HG (2006). Extended leaf longevity in the ore-1 mutant of Arabidopsis with a reduced expression of a plastid ribosomal protein gene. Plant J.

[CR237] Woo HR, Kim HJ, Lim PO, Nam HG (2019). Leaf senescence: systems and dynamics aspects. Annu Rev Plant Biol.

[CR238] Woo HR, Kim HJ, Nam HG, Lim PO (2013). Plant leaf senescence and death - regulation by multiple layers of control and implications for aging in general. J Cell Sci.

[CR239] Woo HR, Koo HJ, Kim J, Jeong H, Yang JO, Lee IH, Jun JH, Choi SH, Park SJ, Kang B, Kim YW, Phee BK, Kim JH, Seo C, Park C, Kim SC, Park S, Lee B, Lee S, Hwang D, Nam HG, Lim PO (2016). Programming of plant leaf senescence with temporal and inter-organellar coordination of transcriptome in Arabidopsis. Plant Physiol.

[CR240] Woodson JD, Joens MS, Sinson AB, Gilkerson J, Salom PA, Weigel D, Fitzpatrick JA, Chory J (2015). Ubiquitin facilitates a quality-control pathway that removes damaged chloroplasts. Science.

[CR241] Wu A, Allu AD, Garapati P, Siddiqui H, Dortay H, Zanor MI, Asensi-Fabado MA, Munné-Bosch S, Antonio C, Tohge T, Fernie AR, Kaufmann K, Xue GP, Mueller-Roeber B, Balazadeh S (2012). JUNGBRUNNEN1, a reactive oxygen species-responsive NAC transcription factor, regulates longevity in Arabidopsis. Plant Cell.

[CR242] Wu H, Li B, Iwakawa HO, Pan Y, Tang X, Ling-hu Q, Liu Y, Sheng S, Feng L, Zhang H, Zhang X, Tang Z, Xia X, Zhai J, Guo H (2020). Plant 22-nt siRNAs mediate translational repression and stress adaptation. Nature.

[CR243] Wu S, Li Z, Yang L, Xie Z, Chen J, Zhang W, Liu T, Gao S, Gao J, Zhu Y, Xin J, Ren G, Kuai B (2016). NON-YELLOWING2 (NYE2), a close paralog of NYE1, plays a positive role in chlorophyll degradation in Arabidopsis. Mol Plant.

[CR244] Wu X, Ding D, Shi C, Xue Y, Zhang Z, Tang G, Tang J (2016). microRNA-dependent gene regulatory networks in maize leaf senescence. BMC Plant Biol.

[CR245] Wu XY, Kuai BK, Jia JZ, Jing HC (2012). Regulation of leaf senescence and crop genetic mprovement. J Integr Plant Biol.

[CR246] Xiao D, Cui Y, Xu F, Xu X, Gao G, Wang Y, Guo Z, Wang D, Wang NN (2015). SENESCENCE-SUPPRESSED PROTEIN PHOSPHATASE directly interacts with the cytoplasmic domain of SENESCENCE-ASSOCIATED RECEPTOR-LIKE KINASE and negatively regulates leaf senescence in Arabidopsis. Plant Physiol.

[CR247] Xiao S, Gao W, Chen QF, Chan SW, Zheng SX, Ma J, Wang M, Welti R, Chye ML (2010). Overexpression of Arabidopsis acyl-CoA binding protein ACBP3 promotes starvation-induced and age-dependent leaf senescence. Plant Cell.

[CR248] Xie Z, Wu S, Chen J, Zhu X, Zhou X, Hoertensteiner S, Ren G, Kuai B (2019). The C-terminal cysteine-rich motif of NYE1/SGR1 is indispensable for its function in chlorophyll degradation in Arabidopsis. Plant Mol Biol.

[CR249] Xu F, Meng T, Li P, Yu Y, Cui Y, Wang Y, Gong Q, Wang NN (2011). A soybean dual-specificity kinase, GmSARK, and its Arabidopsis homolog, AtSARK, regulate leaf senescence through synergistic actions of auxin and ethylene. Plant Physiol.

[CR250] Xu P, Chen H, Cai W (2020). Transcription factor CDF4 promotes leaf senescence and floral organ abscission by regulating abscisic acid and reactive oxygen species pathways in Arabidopsis. EMBO Rep.

[CR251] Xu T, Kim BM, Kwak KJ, Jung HJ, Kang H (2016). The Arabidopsis homolog of human minor spliceosomal protein U11-48K plays a crucial role in U12 intron splicing and plant development. J Exp Bot.

[CR252] Xu X, Bai H, Liu C, Chen E, Chen Q, Zhuang J, Shen B (2014). Genome-wide analysis of microRNAs and their target genes related to leaf senescence of rice. PLoS One.

[CR253] Xu Y, Burgess P, Zhang X, Huang B (2016). Enhancing cytokinin synthesis by overexpressing ipt alleviated drought inhibition of root growth through activating ROS-scavenging systems in Agrostis stolonifera. J Exp Bot.

[CR254] Xu Y, Gianfagna T, Huang B (2010). Proteomic changes associated with expression of a gene (ipt) controlling cytokinin synthesis for improving heat tolerance in a perennial grass species. J Exp Bot.

[CR255] Yamada Y, Furusawa S, Nagasaka S, Shimomura K, Yamaguchi S, Umehara M (2014). Strigolactone signaling regulates rice leaf senescence in response to a phosphate deficiency. Planta.

[CR256] Yamada Y, Umehara M (2015). Possible roles of strigolactones during leaf senescence. Plants.

[CR257] Yamaguchi S (2008). Gibberellin metabolism and its regulation. Annu Rev Plant Biol.

[CR258] Yan H, Saika H, Maekawa M, Takamure I, Tsutsumi N, Kyozuka J, Nakazono M (2007). Rice tillering dwarf mutant dwarf3 has increased leaf longevity during darkness-induced senescence or hydrogen peroxide-induced cell death. Genes Genet Syst.

[CR259] Yang J, Worley E, Udvardi M (2014). A NAP-AAO3 regulatory module promotes chlorophyll degradation via ABA biosynthesis in Arabidopsis leaves. Plant Cell.

[CR260] Yang SD, Seo PJ, Yoon HK, Park CM (2011). The Arabidopsis NAC transcription factor VNI2 integrates abscisic acid signals into leaf senescence via the COR/RD genes. Plant Cell.

[CR261] Yang X, Srivastava R, Howell SH, Bassham DC (2016). Activation of autophagy by unfolded proteins duringendoplasmic reticulum stress. Plant J.

[CR262] Yang Y, Guo Y (2018). Unraveling salt stress signaling in plants. J Integr Plant Biol.

[CR263] Yang Z, Wang C, Qiu K, Chen H, Li Z, Li X, Song J, Wang X, Gao J, Kuai B, Zhou X (2020). The transcription factor ZmNAC126 accelerates leaf senescence downstream of the ethylene signalling pathway in maize. Plant Cell Environ.

[CR264] Yin R, Liu X, Yu J, Ji Y, Liu J, Cheng L, Zhou J (2020). Up-regulation of autophagy by low concentration of salicylic acid delays methyl jasmonate-induced leaf senescence. Sci Rep.

[CR265] Yin XR, Xie XL, Xia XJ, Yu JQ, Ferguson IB, Giovannoni JJ, Chen KS (2016). Involvement of an ethylene response factor in chlorophyll degradation during citrus fruit degreening. Plant J.

[CR266] Yolcu S, Li X, Li S, Kim YJ (2017). Beyond the genetic code in leaf senescence. J Exp Bot.

[CR267] Yoshida S, Ito M, Nishida I, Watanabe A (2002). Identification of a novel gene HYS1/CPR5 that has a repressive role in the induction of leaf senescence and pathogen-defence responses in Arabidopsis thaliana. Plant J.

[CR268] Yoshimoto K, Jikumaru Y, Kamiya Y, Kusano M, Consonni C, Panstruga R, Ohsumi Y, Shirasu K (2009). Autophagy negatively regulates cell death by controlling NPR1-dependent salicylic acid signaling during senescence and the innate immune response in Arabidopsis. Plant Cell.

[CR269] Yu K, Wei J, Ma Q, Yu D, Li J (2009). Senescence of aerial parts is impeded by exogenous gibberellic acid in herbaceous perennial Paris polyphylla. J Plant Physiol.

[CR270] Yuan L, Wang D, Cao L, Yu N, Liu K, Guo Y, Gan S, Chen L (2020). Regulation of leaf longevity by DML3-mediated DNA demethylation. Mol Plant.

[CR271] Zelisko A, Garcia-Lorenzo M, Jackowski G, Jansson S, Funk C (2005). AtFtsH6 is involved in the degradation of the light-harvesting complex II during high-light acclimation and senescence. Proc Natl Acad Sci U S A.

[CR272] Zeng DD, Yang CC, Qin R, Alamin M, Yue EK, Jin XL, Shi CH (2018). A guanine insert in OsBBS1 leads to early leaf senescence and salt stress sensitivity in rice (Oryza sativa L.). Plant Cell Rep.

[CR273] Zentgraf U, Laun T, Miao Y (2010). The complex regulation of WRKY53 during leaf senescence of Arabidopsis thaliana. Eur J Cell Biol.

[CR274] Zhang J, Fengler KA, Van Hemert JL, Gupta R, Mongar N, Sun J, Allen WB, Wang Y, Weers B, Mo H, Lafitte R, Hou Z, Bryant A, Ibraheem F, Arp J, Swaminathan K, Moose SP, Li B, Shen B (2019). Identification and characterization of a novel stay-green QTL that increases yield in maize. Plant Biotechnol J.

[CR275] Zhang K, Gan SS (2012). An abscisic acid-AtNAP transcription factor-SAG113 protein phosphatase 2C regulatory chain for controlling dehydration in senescing Arabidopsis leaves. Plant Physiol.

[CR276] Zhang K, Halitschke R, Yin C, Liu CJ, Gan SS (2013). Salicylic acid 3-hydroxylase regulates Arabidopsis leaf longevity by mediating salicylic acid catabolism. Proc Natl Acad Sci U S A.

[CR277] Zhang K, Xia X, Zhang Y, Gan SS (2012). An ABA-regulated and Golgi-localized protein phosphatase controls water loss during leaf senescence in Arabidopsis. Plant J.

[CR278] Zhang P, Wang WQ, Zhang GL, Kaminek M, Dobrev P, Xu J, Gruissem W (2010). Senescence-inducible expression of isopentenyl transferase extends leaf life, increases drought stress resistance and alters cytokinin metabolism in cassava. J Integr Plant Biol.

[CR279] Zhang S, Li C, Wang R, Chen Y, Shu S, Huang R, Zhang D, Li J, Xiao S, Yao N, Yang C (2017). The Arabidopsis mitochondrial protease FtSH4 is involved in leaf senescence via regulation of WRKY-dependent salicylic acid accumulation and signaling. Plant Physiol.

[CR280] Zhang W, Liu TQ, Ren GD, Hortensteiner S, Zhou YM, Cahoon EB, Zhang CY (2014). Chlorophyll degradation: the tocopherol biosynthesis-related phytol hydrolase in Arabidopsis seeds is still missing. Plant Physiol.

[CR281] Zhang W, Peng KX, Cui FB, Wang DL, Zhao JZ, Zhang YJ, et al. Cytokinin oxidase/dehydrogenase OsCKX11 coordinates source and sink relationship in rice by simultaneous regulation of leaf senescence and grain number. Plant Biotechnol J. 2021;19:335-50. 10.1111/pbi.13467 PMC7868977 33448635

[CR282] Zhang Y, Ji TT, Li TT, Tian YY, Wang LF, Liu WC (2020). Jasmonic acid promotes leaf senescence through MYC2-mediated repression of CATALASE2 expression in Arabidopsis. Plant Sci.

[CR283] Zhang Y, Liu Z, Chen Y, He JX, Bi Y (2015). PHYTOCHROME-INTERACTING FACTOR 5 (PIF5) positively regulates dark-induced senescence and chlorophyll degradation in Arabidopsis. Plant Sci.

[CR284] Zhang Y, Liu Z, Wang X, Wang J, Fan K, Li Z, Lin W (2018). DELLA proteins negatively regulate dark-induced senescence and chlorophyll degradation in Arabidopsis through interaction with the transcription factor WRKY6. Plant Cell Rep.

[CR285] Zhang Y, Wang HL, Gao Y, Guo H, Li Z (2020). SATMF suppresses the premature senescence phenotype of the ATM loss-of-function mutant and improves its fertility in Arabidopsis. Int J Mol Sci.

[CR286] Zhang Y, Wang HL, Li Z, Guo H (2020). Genetic network between leaf senescence and plant immunity: crucial regulatory nodes and new insights. Plants.

[CR287] Zhang Y, Wang Y, Wei H, Li N, Tian W, Chong K, Wang L (2018). Circadian evening complex represses Jasmonate-induced leaf senescence in Arabidopsis. Mol Plant.

[CR288] Zhang Y, Zhao L, Zhao J, Li Y, Wang J, Guo R, Gan S, Liu CJ, Zhang K (2017). S5H/DMR6 encodes a salicylic acid 5-hydroxylase that fine-tunes salicylic acid homeostasis. Plant Physiol.

[CR289] Zhao D, Derkx AP, Liu DC, Buchner P, Hawkesford MJ. Overexpression of a NAC transcription factor delays leaf senescence and increases grain nitrogen concentration in wheat. Plant Biol. 2015;17:904–13. 10.1111/plb.12296 PMC4949518 25545326

[CR290] Zhao MM, Zhang XW, Liu YW, Li K, Tan Q, Zhou S, Wang G, Zhou CJ (2020). A WRKY transcription factor, TaWRKY42-B, facilitates initiation of leaf senescence by promoting jasmonic acid biosynthesis. BMC Plant Biol.

[CR291] Zhao Y, Chan Z, Gao J, Xing L, Cao M, Yu C, Hu Y, You J, Shi H, Zhu Y, Gong Y, Mu Z, Wang H, Deng X, Wang P, Bressan RA, Zhu JK (2016). ABA receptor PYL9 promotes drought resistance and leaf senescence. Proc Natl Acad Sci U S A.

[CR292] Zhou C, Cai Z, Guo Y, Gan S (2009). An Arabidopsis mitogen-activated protein kinase cascade, MKK9-MPK6, plays a role in leaf senescence. Plant Physiol.

[CR293] Zhou C, Han L, Pislariu C, Nakashima J, Fu C, Jiang Q, Quan L, Blancaflor EB, Tang Y, Bouton JH, Udvardi M, Xia G, Wang Z-Y (2011). From model to crop: functional analysis of a STAY-GREEN gene in the model legume Medicago truncatula and effective use of the gene for alfalfa improvement. Plant Physiol.

[CR294] Zhou H, Zhao J, Cai J, Patil SB (2017). UBIQUITIN-SPECIFIC PROTEASES function in plant development and stress responses. Plant Mol Biol.

[CR295] Zhou J, Lu D, Xu G, Finlayson SA, He P, Shan L (2015). The dominant negative ARM domain uncovers multiple functions of PUB13 in Arabidopsis immunity, flowering, and senescence. J Exp Bot.

[CR296] Zhou X, Jiang Y, Yu D (2011). WRKY22 transcription factor mediates dark-induced leaf senescence in Arabidopsis. Mol Cells.

[CR297] Zhu JK (2016). Abiotic stress signaling and responses in plants. Cell.

[CR298] Zhu K, Tao H, Xu S, Li K, Zafar S, Cao W, Yang Y (2019). Overexpression of salt-induced protein (salT) delays leaf senescence in rice. Genet Mol Biol.

[CR299] Zhu X, Chen J, Xie Z, Gao J, Ren G, Gao S, Zhou X, Kuai B (2015). Jasmonic acid promotes degreening via MYC2/3/4-and ANAC019/055/072-mediated regulation of major chlorophyll catabolic genes. Plant J.

[CR300] Zhuang XH, Jiang LW (2019). Chloroplast degradation: multiple routes into the vacuole. Front Plant Sci.

[CR301] Zhuo M, Sakuraba Y, Yanagisawa S (2020). A Jasmonate-activated MYC2-Dof2.1-MYC2 transcriptional loop promotes leaf senescence in Arabidopsis. Plant Cell.

[CR302] Zwack PJ, De Clercq I, Howton TC, Hallmark HT, Hurny A, Keshishian EA, Parish AM, Benkova E, Mukhtar MS, Van Breusegem F, Rashotte AM (2016). Cytokinin response factor 6 represses cytokinin-associated genes during oxidative stress. Plant Physiol.

[CR303] Zwack PJ, Rashotte AM. Cytokinin inhibition of leaf senescence. Plant Signal Behav. 2013;8:e24737. 10.4161/psb.24737 PMC3908980 23656876

